# The Animal-Visitor Interaction Protocol (AVIP) for the assessment of *Lemur catta* walk-in enclosure in zoos

**DOI:** 10.1371/journal.pone.0271409

**Published:** 2022-07-28

**Authors:** Ilaria Pollastri, Simona Normando, Daniela Florio, Linda Ferrante, Francesca Bandoli, Elisabetta Macchi, Alessia Muzzo, Barbara de Mori

**Affiliations:** 1 Department of Comparative Biomedicine and Food Science, University of Padua, Legnaro, PD, Italy; 2 Ethics Laboratory for Veterinary Medicine, Conservation and Animal Welfare, University of Padua, Legnaro, PD, Italy; 3 Department of Veterinary Medical Science, University of Bologna, Ozzano dell’Emilia, BO, Italy; 4 Giardino Zoologico di Pistoia, Pistoia, Italy; 5 Department of Veterinary Science, University of Turin, Grugliasco, TO, Italy; Sichuan University, CHINA

## Abstract

Animal–Visitor Interactions (AVI) are activities offered by zoos and other tourism facilities, in which visitors come into close contact with animals. These activities can promote conservational and educational content, raise conservation mindedness and responsibility for the environment and animal welfare, but if not properly managed can jeopardize visitors’ and animals’ well-being and conservation efforts. The Animal-Visitor Interaction assessment Protocol (AVIP) has been designed to perform an integrated and multidisciplinary assessment of these activities, encompassing the “One Health, One Welfare” approach. AVIP throughout six different steps allows to assess the effects of AVIs both on animals, visitors, and the staff involved. Results can assist zoos to improve management decisions, ensure a transparent evaluation of their activities and promote conservation education goals. Lemurs walk-in enclosures have become increasingly popular among zoos, nevertheless studies focused on their assessment are still scarce. To validate AVIP to this particular AVI, we applied it to assess a walk-in enclosure hosting five *Lemur catta* in an Italian zoo. Results of behavioural and physiological analyses suggested no changes in animal welfare level and the Animal Welfare Risk Assessment showed low animal welfare risks. Two Visitor Experience Surveys were used to interview 291 visitors, showing that the assessed AVI could help promote the zoo’s conservation objectives and visitor education. Risk Assessment found low and medium risks to the health and safety of visitors. Results were then combined to perform a final ethical assessment. Some potential ethical concerns were detected, but the outcomes indicated that these conflicts were well managed. In the context of recent findings AVIP demonstrated its potential for application also in assessing AVIs involving primates. Our findings confirmed the usefulness of AVIP in assessing and monitoring AVIs, allowing to gain key information in a single process on multiple welfare-related parameters, educational impact, safety of the main stakeholders involved, and ethical concerns.

## Introduction

Animal-Visitor Interactions (AVIs) are very common in modern zoos and aquaria worldwide [1]. Recent studies show that AVIs can be a powerful way of maximizing both education and memorable experiences [2,3], increasing positive attitudes of zoo visitors towards natural and conservation issues, and facilitating the multiple goals of modern zoos regarding conservation, education, research, and animal welfare [1]. AVIs include animal presentations, behind the scenes encounters, close-up encounters, and walk-in enclosures with free-ranging animals [4,5].

Despite the requirements provided by the Guidelines of the World Association of Zoos and Aquaria (WAZA) [6,7], the overall impact that AVIs can have on animal welfare, health, and safety, as well as on visitors’ experience, conservation educational outcomes, and safety, are still not assessed on a regular basis [8]. Although some studies exist (as reviewed by [9–12], scientific research on these impacts is still needed due also to the wide range of species involved and the considerable variability of AVIs [1,8]. To date, studies have shown that involving live animals in educational and entertainment activities in zoos, such as AVIs, not only can increase educational opportunities, but can be useful in changing visitor attitudes regarding wildlife, as well as rising conservation mindedness and responsibility for the environment [9–14]. Also the effects that zoo visitors can have on the welfare of captive animals have been the focus of recent scientific interest [15–17], showing that presence and density of visitors, as well as their behaviour, may have an impact on animal welfare. This impact can be either negative (undesirable), positive (enriching), or merely a changing variable that has no effect [17–22]. However, studies have also shown that AVIs not properly managed can lead to welfare problems for the animals involved [2,23], transmit erroneous conservational and educational messages (e.g., perceive wild animals to be suitable pets), have detrimental impacts on the conservation of the species involved (e.g., wildlife illegal trade), thus jeopardizing the zoo’s mission [2]. Therefore, due to the increasing number of AVIs provided in zoos, it has become especially important to assess their impact both on animals, visitors and staff involved [16].

**Fig 1:**
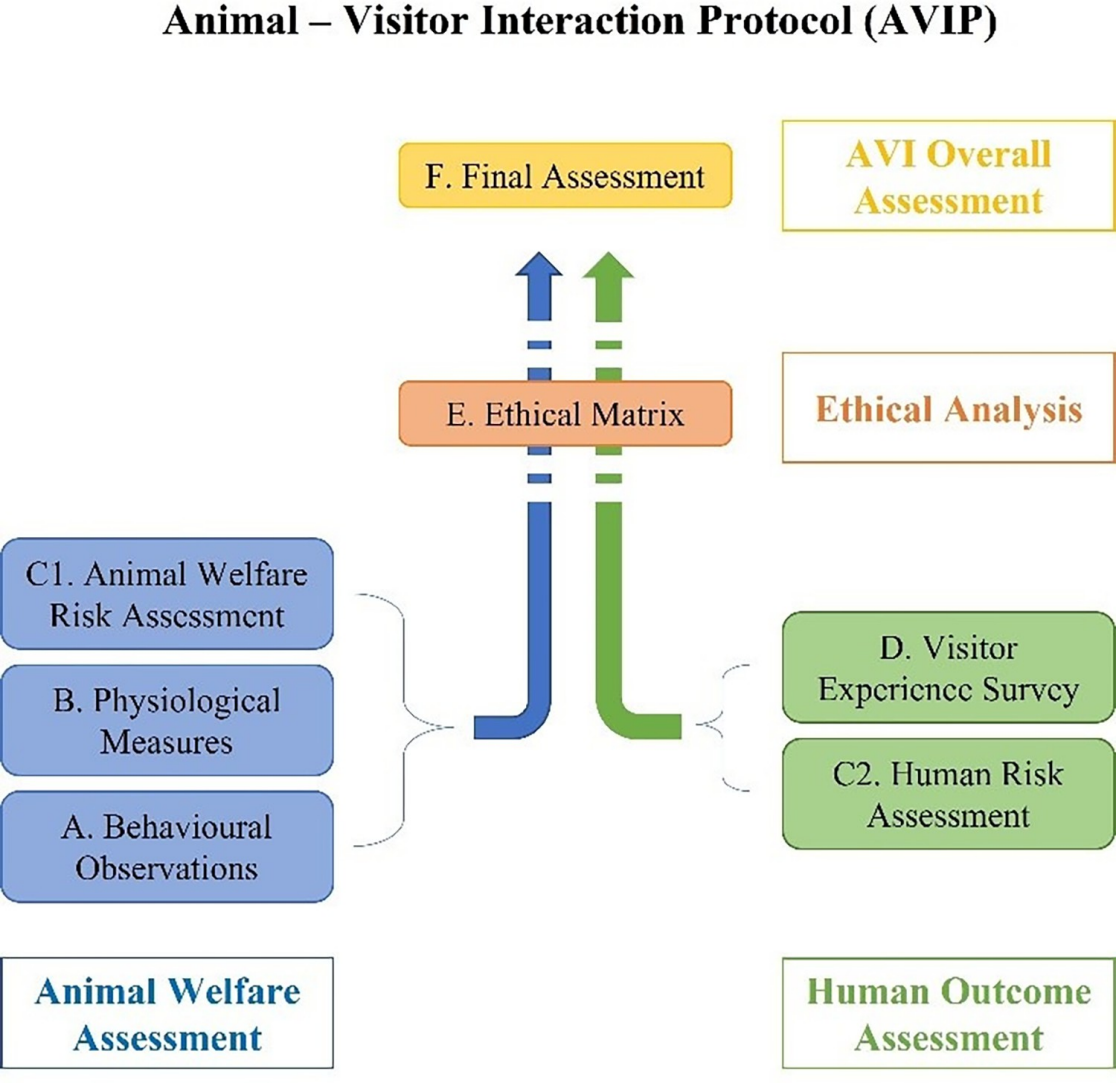
Description of the Animal-Visitor Interaction Protocol (AVIP). The behavioural (step A) and physiological (step B) assessments, together with the Animal Welfare Risk Assessment (step C1), enable to evaluate the effects and consequences of AVIs on animal welfare and health. Steps from C2 to F allow to assess the impact of AVIs on humans involved and to make a final evaluation: Step C2 allows evaluating the impact of AVIs on the safety and welfare of visitors, while step D investigates changes in visitors’ attitudes towards animals and conservation issues, as well as their education and experience in terms of motivation and expectations. The ethical assessment of AVIP (step E) enables to interpret and discuss results obtained by comparing them with an Ethical Matrix, representing the ideal situation for all stakeholders. Finally, through the final checklist (step F), it is possible to provide an explicit result of the evaluation process, by which strengths and weaknesses of an AVI can be identified, managed and communicated. Since both the variation in zoo animal responses to visitors and visitor experience may be the result of species and situation-specific differences, individual animal characteristics, enclosure design, or AVIs nature, the AVIP protocol should be adapted every time to the specific context of the AVI to be evaluated. As a result, AVIP can help to improve management decisions and to ensure a transparent evaluation of AVI activities.

Following WAZA recommendations [6,7] and due to the different kinds of AVIs proposed in zoos worldwide [1], an Animal-Visitor Interaction assessment Protocol (AVIP) has been designed to perform an integrated and multidisciplinary assessment of these activities [24]. Thanks to its multi-disciplinary approach, AVIP encompasses the ’One Health, One Welfare’ concept, which recognizes that many aspects of animal well-being are intrinsically linked to those of humans [25]. Throughout six different steps, AVIP allows to assess the effects of AVIs both on animals, visitors and the staff involved (Fig 1).

In zoos, walk-in enclosures AVIs have become increasingly common [26,27] as they allow a particular kind of AVI where visitors experience close proximity to wild captive animals without physical barriers. There is evidence that the closeness generated by this kind of setting is preferred by visitors, as they can see animals in a more naturalistic way [28]. Studies found this kind of encounters exert positive effects on visitor attitude, like increasing interest towards the animals and developing a connection with the wild ones [29]. For example, Price et al. (1994) highlighted that visitors’ reactions to free-ranging cotton-top tamarins (*Saguinus oedipus*) were more favorable compared to the reactions to caged tamarins. Indeed, visitors spent more time watching them, made more comments about them, and perceived them to have improved welfare and a higher educational value than their caged counterparts [30]. Nonetheless, research recently showed that negative impact on animals could occur in some scenarios. This has been observed particularly in walk-in enclosures with shy species or individuals, if they are unable to avoid unfamiliar visitors, sights, and sounds [1]. For example, Learmont et al. (2018) found that quokkas (*Setonix brachyurus*) were less visible in their preferred areas when visitors were present, indicating that visitors were at least moderately fear-provoking for that group of animals [31]. Similarly, Larsen et al. (2014) discovered that koalas (*Phascolarctos cinereus*) spent more time being vigilant as the number of visitors and noise increased [32]. Sherwen et al. (2015) noted that western grey kangaroos and red kangaroos (*Macropus fuliginosus fuliginosus*, and *Macropus rufus*) showed not only an increase of time spent in vigilant behaviour and locomotion, but also a decrease in time spent resting [26]. Moreover, the kangaroos increased the time spent in retreat areas when visitors were present [26]. This notwithstanding, research on the effects of these walk-in enclosures on animal welfare is still limited [26].

Non-human primates are commonly housed in walk-in enclosures. In particular, lemur walk-in enclosures have become increasingly popular in zoos, as viewing lemurs has been found to be attractive to visitors [28,33–35]. However, even if research on non-human primate behaviour, welfare, and visitor effects is vast, scientific evidence of the impact of walk-in enclosures on their overall welfare is limited to few species. For example, Jones et al. (2016) found that allowing visitors into the exhibit of crowned lemurs (*Eulemur coronatus*) had a positive effect by decreasing lemur aggression [36]. Studies on lemurs, in particular *Lemur catta*, have shown the suitability of this species to this type of enclosure. Manna et al. (2007) found that the ring-tailed lemurs (*Lemur catta*) housed in a walk-in exhibit were more active and spent less time resting when visitors were present within the enclosure [37]. Collins and colleagues reported that behaviour and behavioural diversity of ring-tailed lemurs at Fota Wildlife Park (Ireland) were little affected by visitors, highlighting their habituation to them [38]. According to Goodenough et al. (2019), the time of day and weather (which vary with visitor numbers) have a negative impact on the behaviour of a group of 19 ring-tailed lemurs housed in a walk-in enclosure at West Midlands Safari Park (Worcestershire, UK) [28]. However, they showed that this impact had a greater influence on the animals’ behaviour than the presence of visitors in the enclosure. Furthermore, the advantages of walk-in enclosures in terms of visitor experience and education outweigh the negative effects of visitors on animal behavior. Similarly, Farhall et al. (2010) found that the behaviour of nine lemurs housed in a walk-in enclosure at Cotswold Wildlife Park (CWP) was significantly influenced by the weather but not by time or visitor presence, whereas they showed that enclosure use was affected by weather, time, and visitor presence [39]. Conversly, Snipp (2004) found that visitor presence within a walk-through lemur exhibit had little influence on the behaviour and exhibit use of the animals [40]. Also Hosey et al. (2016) reported that the visitor presence in a lemur walk-in enclosure did not affect the animals’ behaviour, and that the levels of lemur–lemur wounding did not correlate with the number of people in the zoo [41]. However, as animals’ reactions to visitors varied among the species and zoos, in AVI assessment, it is important to consider the temperament, adaptation to the surroundings, and characteristics of the species and the individuals involved. For example, in some cases, visitors’ access may be restricted (so that they cannot touch, feed or approach the animals too closely) or may be without control, representing a variable that could affect the animals.

The present study is focused on AVIP application on a specific AVI involving a group of ring-tailed lemurs (*Lemur catta*) housed in a walk-in enclosure at the Giardino Zoologico di Pistoia (Pistoia, Italy). During the interaction, Giardino Zoologico di Pistoia’s visitors are allowed to enter the lemur enclosure and have a close encounter with the animals while receiving information about them. Therefore, each step of the AVIP protocol [24] was customized to focus on the specific features of the AVI under evaluation. AVIP is different from other evaluation methodologies of AVIs, in that it allows at the same time to gain key information on multiple welfare-related parameters, analyse the educational impact, the safety of the main stakeholders involved, and ethical concerns in one protocol, following WAZA guidelines and a "One Health, One Welfare" approach. Although the obtained results refer to a specific situation, they are discussed in the context of recent findings regarding visitor presence, zoo animal welfare, and behavioural research on primates in zoos. Our findings confirmed the usefulness of AVIP in assessing and monitoring AVIs, also when involving primates.

## Materials and methods

### Ethical approval

The study was performed in full compliance with the Guidelines for the Treatment of Animals in Behavioural Research and Teaching (2006) and with the WAZA Code of Ethics and Animal Welfare (2003), and with authorization from the Giardino Zoologico di Pistoia (www.zoodipistoia.it), Pistoia, Italy. This zoological institution is accredited by the EAZA (European Association of Zoos and Aquaria) and UIZA (Unione Italiana Giardini Zoologici e Acquari) and it has rigorous standards for animal welfare. Zoological gardens in Italy are expected to perform behavioral observations of the individuals in their care (D. Lgs.73/2005). Since all recording procedures were non-invasive and the husbandry routines of the animals involved were not changed or affected by the study, this study does not fall in any of the categories for which approval of an ethic committee is required by Italian laws (D.Lgs. 26/14, implementation of the Directive 2010/63/EU).

Informed consent was obtained from all the surveys participants. A privacy notice was provided in the survey to inform and assure that responses were anonymous, confidential, and that information collected would be used for research purposes only. No personal data were collected. Participation was voluntary and could be cancelled at any time without any reason. No financial or gift compensation was proposed for completing the survey. The participation in the surveys did not interfere with the day at the zoo or the daily life of participants.

### Animals, housing, and husbandry

The animals involved in the present study were a group consisting of five ring-tailed lemurs (*Lemur catta*), all born at Giardino Zoologico di Pistoia: Sakalava born in 2006, Mandrare born in 2001, Bekili and Andribe born in 2000 and the only male housed, Ankarana, born in 2005. All five individuals were related. Lemurs were housed together in an enclosure (“Voliera dei lemuri”) consisting of an indoor area, not visible to visitors, that opens to an external area. The internal area, of 15 m^2^, 3 m high, consisted of two rooms equipped with branch platforms, a nest box, and heating. The external area was 200 m^2^, 5 m high, and delimited by a nylon net. It can be divided into two different parts: a lower artificial area, consisting of a cemented path used for the transit of the keepers and visitors inside the enclosure, and a 1 m raised area consisting of soil with natural vegetation (trees, shrubs, and grass). In this second part of the external enclosure, natural and artificial supports were placed at different heights. In addition, on two sides of the outdoor area, there were doors used for the entrance and exit of the public. From the outside of the enclosure, visitors could see inside through two glass-walls, one placed in the middle of the enclosure and the other one at the end (S2 Fig).

Animals were free to access the indoor area all day. Moreover, in wintertime and until the weather temperatures become milder, the internal area was heated.

Feeding provisions took place twice a day, in the morning and evening. Fruits and seasonal vegetables were given to the lemurs into feeding bowls located in the internal area. Water was provided ad libitum in both areas of the enclosure. Moreover, leaves, flowers, and berries were naturally present in the enclosure and always available to the animals. Enclosures were cleaned daily in the morning.

### The animal-visitor interaction: “A tu per tu con i lemuri”

At the Giardino Zoologico di Pistoia, visitors have the possibility to enter the outside area of the lemurs’ enclosure for a closely encounter with the animals. This interactive activity, “A tu per tu con i lemuri,” differs depending on whether it is a weekday or weekend day. Over the weekend (Saturday, Sunday, and public holidays), the interaction is scheduled from two to four times a day. An educator opens the enclosure to the public, and small groups of on average six people are guided in the external area of the enclosure, where the activity takes place. During the activity, the educator presents some information about lemurs (geographical distribution, behaviour, social system, conservation status, the zoo’s conservation projects, etc.). When the talk finishes, and visitors have no more questions, the interaction ends, and the educator leads the visitors to the exit. Each session lasts around 5/10 minutes.

During the weekday, the activity occurs differently. In those days, the lemur’s enclosure is opened by an animal keeper only once a day for 20 minutes in the afternoon. No information about the animals is given to visitors, and on average six visitors can access the enclosure at the same time. Once inside, they can stay there as long as they wish or depending on the number of people waiting outside.

In both situations, the opening and closing of the gates are always regulated by the zoo staff, and only happens in the presence of them. In their absence, public can only observe the lemurs from the outside, through glass—walls. Furthermore, before entering the enclosure, visitors receive information on behavioural rules to be respected, and posters indicating the timing, regulation, obligations, and prohibitions are shown (e.g., to stay in the artificial part of the enclosure, not to touch the animals, not to smoke, etc.). Moreover, visitors are asked to leave strollers and food out of the enclosure, to disinfect shoe outsoles, and to cleanse their hands with disinfectant gel provided by the keepers both at enclosure entrance and exit. The zoo staff checks that the rules are respected, and controls that visitors maintain a minimum distance of one meter from the lemurs. During the interaction, the animals are observed without requiring them in any way to stay close to the visitors.

### AVIP procedures and data collection

The aforementioned AVI "A tu per tu con i lemuri" was assessed in August and September 2018 at the Giardino Zoologico di Pistoia (Pistoia, Italy—43°55’46.6"N 10°51’59.7"E). To carry out the evaluation of “A tu per tu con i lemuri” AVI, some details of the AVIP methods previously described for a giraffe feeding AVI [24,42] were adapted to the new context. Therefore, S1 Table describes the adaptations applied for the first five steps (A, B, C, D, E), while step F (i.e., the final checklist) was instead not modified from [24].

Animals were recorded in four different situations: during weekday afternoon activity (EEK), during weekend morning activity (EE1) and weekend afternoon activity (EE2). Moreover, on weekday morning, a control session (CON) was carried out, following the same recording timing of EE1 (no longer than 20 minutes). In these different situations, the animals were recorded before, during and after the AVI (S1 Fig). Animals were recorded using continuous focal animal sampling [43,44] and behaviours were analyzed using a working ethogram (S2 Table). The ethogram was adapted from previous studies [45–54] and finalized during a one-week period of preliminary observations using ad lib observations, as defined by [43]. Moreover, during the one-week preliminary period, the observer practiced the recognition of individual animals, thanks to specific signs made with temporary hair dye (BioKap® Spray Ritocco, Bios Line S.p.a. (PD)).

During observations, the researcher was supposed to record any abnormal behavior, any attempt of the animals to avoid contact/close proximity with interacting visitors, and any escape attempt. Also, the researcher was instructed to record any other behavioral pattern not highlighted by the preliminary observations but deemed of potential interest.

The lemurs’ enclosure was virtually divided into several areas (S2 Fig): areas 2, 4, and 6 correspond to the cemented path used for people transit inside the enclosure; areas 1, 3, and 5 correspond to the natural part of the enclosure, not accessible to visitors, in which natural and artificial supports allow the lemurs to use the space vertically; RI correspond to the night enclosure. During each recording session, the position of the animals in each area was recorded.

The lemurs’ individual faecal samples were collected in the enclosure, stored at -20°C, and then analysed to detect any changes in levels of faecal glucocorticoid metabolites (FGM) between periods of low density of visitors (during the weekday) and periods of high density of visitors (during the weekend). To measure the cortisol level matched to the recorded day, the samples were collected the following day, as there is a time interval between the release of cortisol and FGM excretion in the faeces [55,56].

The information gathered by the two previous steps was used to perform an Animal Welfare Risk Assessment. Following the methodology provided by the EFSA guidelines [57], through a series of phases it is possible to identify any risk for animal welfare and to calculate a Welfare Score, as described in S1 Table.

Moreover, for each recorded interaction day, a Visitor Experience Survey was administered after the AVI to the participants. On the same days, a second Visitor Experience Survey was delivered near the zoo exit to visitors who either attended or did not to the AVI programs.

Visitor Safety Risk Assessment was performed following the Department for Environment, Food, and Rural Affairs (DEFRA) [58] and EAZA [59] documents. This step aims to highlight possible risks for visitors’ safety and health, identify measures to eliminate or reduce hazard exposure, and examine the existing prevention and protection measures. It also allows to find preventive and protective actions that can be implemented to ensure the protection of the visitors during AVIs. Therefore, through several phases, it is possible to calculate the risk, defined as the probability that a hazard occurs multiplied by the magnitude of the damage that can derive from the hazard if it happens.

To highlight possible ethical concerns, results obtained from the previous A-D Steps were analyzed using an Ethical Matrix (EM) [60]. The Ethical Matrix is a conceptual tool that frames the ethically relevant demands in a complex situation where several moral conflicts are difficult to address. The EM’s purpose is to summarize all the ethical demands, in terms of needs and interests involved in a given situation, of the relevant stakeholders allowing decision-makers to analyze the different points of view and map potential ethical conflicts. The EM consists of a table where the rows list the three main ethical principles of common morality (well-being, autonomy, fairness), and the first column lists the various stakeholders involved. All the cells of the EM are filled with specific moral demands. In AVIP, the EM is tailored to represent the ideal situation of the AVI under scrutiny for all stakeholders (Step E).

Finally, through the Step F final checklist, strengths and weaknesses of the AVI are identified, allowing be sharing and communicating.

### Faecal sample analyses

As recommended, to extract steroids from nonliquid matrices (such as dried solids), faeces were subjected to an organic phase extraction using ethanol [61]. Extraction and determination of FGM were carried out as previously reported by [62]. Following the protocol, faecal samples were kiln dried at 55°C for 24 hours, thoroughly crushed, and five aliquots of pulverized feces (0.20 g each) were put into extraction tubes, which were then sealed with a Teflon cap (Pechiney Plastic Packaging Inc., Menasha, WI). Next, 1 mL of ethanol (Sigma-Aldrich, St. Louis, MO) for every 0.1 g of solid was added to each tube, and the mixture was shaken vigorously for 30 minutes. Samples were centrifuged at 3,300g for 15 minutes, and the supernatant was recovered in a clean tube for evaporation to dryness in a SpeedVac (Thermo Fisher Scientific, Waltham, MA). Extracts were stored at -80°C. Extracted samples were dissolved in 100 mL of ethanol followed by at least 400 mL of kit Assay Buffer (Arbor Assays, Ann Arbor, MI); then, they were vortexed and rested for 5 minutes twice, to ensure complete steroid solubility. FGM was determined using a multi-species cortisol enzyme immunoassay kit (K003; Arbor Assays, Ann Arbor, MI) validated for dried faecal extracts. All analyses were repeated twice. It is uncertain to which extent native molecules and immunoreactive metabolites of cortisol were quantified in the kit used. As a consequence, the terminology faecal glucocorticoid metabolites were used. Inter-assay and intra-assay coefficients of variation were less than 10%. The test’s sensitivity was determined by measuring the least amount of hormone standard consistently distinguishable from the zero-concentration standard and was calculated to be 24.5 pg/mL.

According to the manufacturer, the cortisol kit presents the following cross-reactivity: 100% with cortisol, 18.8% with dexamethasone, 7.8% with prednisolone, 1.2% with corticosterone, and 1.2% with cortisone. Serial dilutions (1:4, 1:8, 1:16, and 1:32) of faecal samples were assayed to test for parallelism against the standard curve (P < 0.05 for all assays). The mean recovery rate of cortisol added to dried feces was 97.2%.

### Statistical analysis

#### Behavioural observations (Step A)

A first analysis was performed on the quantity of “Not visible”. Since the length of the “Not visible” periods (the time the animals were not visible) was not negligible relative to the length of the observed behavioral patterns to be recorded, the time the animals were not visible was deleted from the sample, reducing the sample period accordingly, following Lehner 1996, p. 193 [63] for subjects disappearing from view. As a consequence, data for states were represented as the ratio between the duration of each behavior in a given session and the time in which the animals were visible in the same session. Similarly, event’s frequency was calculated as the number of occurrences of the behavioural pattern in a given session divided by the number of minutes the animal was visible in the same session. Then statistical analyses were run, both on the five lemurs as a group, and at the individual level (as advised by [15,64]), by comparing the observations made in analogous sessions (e.g., “pre-” vs. “pre-”) (as in [42,65]). Therefore, behaviours performed in the ten control sessions (CON) were compared with the ten weekend morning interactions (EE1), and the behaviours observed in the eight weekday afternoon interactions (EEK) were compared with the eight weekend afternoon interactions (EE2). The Shannon-Weiner diversity index H [66], also referred to as Entropy [67] was calculated to investigate the behavioural diversity of the lemur group on the visible time and the enclosure use on the total time. The decision to calculate the latter on total time and the former on visible time was taken because, during observations, it had been always possible to know where an animal was, but not to always discriminate what behaviour the animal was performing (e.g., only a part of the animal was seen; the animal was not outside, so he/she was inside).

The index has been calculated using the following formula:

$$H=-\sum (p_{i}\;\log p_{i})$$

where p is the proportion of time engaged in the behaviour of the ith. Higher values indicate greater behavioural diversity or even enclosure use. The H index of analogous sessions (e.g., “pre-” vs. “pre-”) were compared following the previous comparisons (CON vs EE1 and EEK vs EE2).

Finally, the ambient temperatures recorded were compared between control and interaction episodes to verify that they did not differ, as this could be a source of bias for the results.

All the comparisons were made using the Mann-Whitney U-test. Even if no data set was used twice in the comparisons, Bonferroni correction for multiple comparisons was applied in order to be more rigorous. Therefore, behaviours are considered to significantly differ if the *p-*value was less than 0.025 (0.05/2 because there are two comparisons). Differences with *p-*value between 0.05 and 0.025 are considered as tendencies. All statistical analyses were performed using the software IBM SPSS Statistics 21.0 (IBM Corp, Armonk, NY).

#### Physiological measures (Step B)

The samples of each individual were analyzed to detect differences in FGM concentration between periods of low density of visitors (during the weekday–“LOW”) and periods of high visitor density (during the weekend–“HIGH”). Student t-test was performed with alpha set at 0.05. The statistical analyses were performed using the software IBM SPSS Statistics 21.0 (IBM Corp, Armonk, NY).

#### Visitor experience survey (Step D)

A Chi‐squared contingency test was done to investigate whether groups (PostQ vs. GenQ) or respondents who joined an interaction or who did not join an interaction have distinctive characteristics (sex, educational level, pet ownership, natural childhood, first visit, age). Independent samples t-test was used to investigate the Likert scale statements. The analysis of visitor satisfaction was investigated with the Kano Model: the answers of each respondent to the functional and dysfunctional questions of each attribute were classified by the combination of the answers. For each attribute, the combination chosen by the majority of the respondents defined the classification of the attribute: Exciter, Indifferent, Questionable response, Reverse, Must-have, Linear. The frequency of respondents choosing Linear, Exciter, Must-have and Indifferent were used to calculate the customer satisfaction and dissatisfaction coefficient (CS) (see [24,68,69] for further details about the procedure).

All statistical analyses were performed using the software IBM SPSS Statistics 21.0 (IBM Corp, Armonk, NY).

## Results

### Animal welfare assessment (Step A to C1)

#### Behavioural observations (Step A)

The statistically significant differences detected during the group comparisons CON vs EE1 and EEK vs EE2 of analogous sessions (e.g., “pre-” vs. “pre-”) carried out when the animals were visible are reported in Table 1. No differences were detected in ambient temperatures in the comparison between control and interaction episodes. Results of the analyses performed on behaviours at the individual level (as advised by [64]) are reported as supplementary material (S3 Table).

**Table 1:** Medians, interquartile range of the behaviours which resulted significantly different among sessions and Mann-Whitney U-test results. Behaviours in *italics* indicate events. Only behaviours which significantly differed or showed a tendency to differ are reported. *P*-values not reported in bold are those which after Bonferroni correction are not significant but represent a tendency.

Behaviour	Session	Median	IQR	Mann-Whitney U-test	P-value
Self-grooming	CON–pre	0	0.05	U = 924	**p = 0.012**
	EE1 –pre	0.02	0.21		
Eating natural food	CON–pre	0	0	U = 1071	**p = 0.023**
	EE1 –pre	0	0		
Huddling	CON–dur	0	0.7	U = 883	**p = 0.004**
	EE1 –dur	0.27	1		
Mutual licking	CON–dur	0	0	U = 1067	p = 0.031
	EE1 –dur	0	0		
Sunning	CON–post	0	0	U = 1150	p = 0.042
	EE1 –post	0	0		
*Urinate*	CON–post	0	0	U = 1125	**p = 0.023**
	EE1 –post	0	0		
Eating natural food	EEK–dur	0	0	U = 680	**p = 0.011**
	EE2 –dur	0	0		
Huddling	EEK–post	0	0	U = 635.5	p = 0.047
	EE2 –post	0	0.68		
Grooming by conspecific	EEK–post	0	0	U = 720	p = 0.042
	EE2 –post	0	0		
*Sniffing*	EEK–post	0.18	0.32	U = 573.5	**p = 0.012**
	EE2 –post	0	0		
*Yawn*	EEK–post	0	0	U = 720	p = 0.042
	EE2 –post	0	0		

The Mann-Whitney U test detected a statistically significant difference in the behavioural diversity index, showing that during the pre-session of the control the animals performed more *state* behaviours (or more animals performed different behaviours) than during the pre-session of the morning weekend interaction (CON-pre: H index ranging from 0 to 0.75; EE1-pre: H index ranging 0 to 0.71; U = 845; p = 0.005).

Overall enclosure use was higher on the dur-session of the control compared to the dur-session of the weekend morning interaction (CON-dur Entropy index ranging from 0 to 0.47; EE1-dur Entropy index ranging from 0 to 0.40; U = 927.5, p = 0.01). No other differences were detected. Analyzing the proportion of time the animals spent in the different areas in the comparison CON-dur vs EE1-dur, the Mann-Whitney U test detected that the animals spent more time in the internal area of the enclosure during the during session of the control that the during session of the weekend morning interaction (RI-CON-dur median = 0; IQR = 0.42; RI-EE1_dur median = 0; IQR = 0; U = 929, p = 0.002).

#### Physiological measures (Step B)

**Fig 2:**
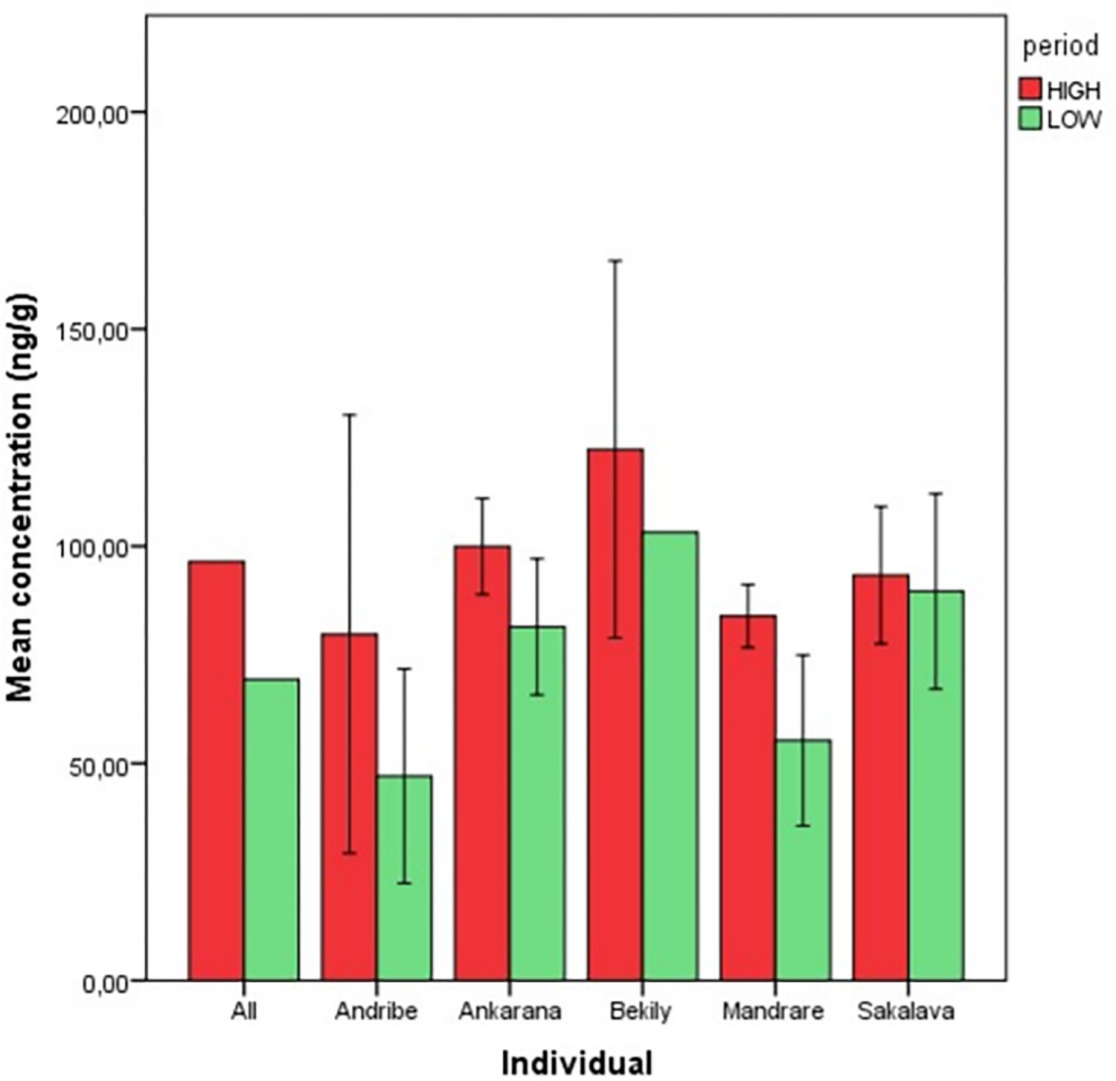
Mean concentrations of faecal cortisol metabolites (FCM) of the five lemurs during the “LOW” and “HIGH” sampling. Mean value ± Standard error are reported.

We analyzed a total of 41 faecal samples, 19 collected in “LOW” sampling days, and 22 in “HIGH” sampling days. Measured FGM ranged from 16.88 to 173.95 ng/g feces. The mean FGM concentration values were lower in the "LOW" sampling days (mean ± SE: 69.320 ± 6.621 ng/g) than in the "HIGH" periods (mean ± SE: 96.532 ± 5.952 ng/g) for all the subjects. Analysing the mean FGM concentration, the statistical test did not detect any significant difference between “LOW” sampling days and “HIGH” sampling days for any of the five lemurs (Fig 2).

#### Animal risk assessment (Step C1)

Results obtained by the previous steps were integrated in the Animal Welfare Risk Assessment.

*Problem formulation phase—Identification of factors of animal welfare concern*. The “Management Checklist: Staff action and Procedures” (Table A in S1 Appendix) did not record negative answers, showing that the management and enclosure were adequate to maintain a high standard of welfare during the interactions.

*Welfare risk assessment—Exposure assessment*. Scenario 1 and scenario 2 have different consequences (behavioural changes that indicate that the animals were at risk of perceiving negative subjective experiences and injuries) while recognizing the same exposure factors (improper approach of the visitor). Based on the analysis of the video recordings of the during-sessions (dur-EE1, dur-EE2 and dur-EEK), the frequency of exposure was identified as negligible. For scenario 3, a low probability of effective contacts was defined. Even when visitors wash and disinfect their hands before the interaction activity, the spreading of some pathogens by droplets or aerosol may be possible in the absence of preventive measures. No clinical signs attributable to infectious or diffusive disease were observed during the study. Moreover, all periodical clinical examinations performed by the zoo veterinarian, which were supported by laboratory tests for specific zoonotic agents, had negative outcomes. Nonetheless, the probability of disease spreading was considered low and not negligible as a precautionary measure.

*Welfare risk assessment–Consequence characterization*. In our study, no consequences on animal welfare were observed, but as a precautionary measure, the probability has been defined as low and not as negligible. We cannot exclude, due to the short period of observation, the possibility that there could be negative long-term consequences for welfare.

*Welfare risk assessment–Risk characterization*. The WS value was calculated for each exposure scenario. The results are presented in the following table (Table 2).

**Table 2:** Welfare score values (FE = frequency of exposure; FC = frequency of consequences; MA = magnitude of consequences; WS = Welfare Score). Adapted from [42].

Exposure Assessment		Consequences Characterization				Risk Characterization
Hazard Description	FE	Animal-Based Indicators	Severity	Duration	FC	WS = FE * MA * FC
Improper approach (scenario 1)	1	Behavioural observation	1	1	2	2
Improper approach (scenario 2)	1	Skin Lesion	1	1	2	2
Effective contact with a zoonotic agent (scenario 3)	2	Zooanthroponosis	1	1	2	4

### Human outcome assessment (Steps C2 and D)

#### Human risk assessment (Step C2)

The “Management Checklist: Preventive and Protective Measures” (Table A in S2 Appendix) applied for the analysis of the preventive and protective measures needed to guarantee adequate safety standard of people during interactions recorded eight negative answers. The items concerned:

- The existence of a safe area, and thus the possibility to store personal item in the safe area (5.5., 1.2)
- The existence of a service access point differentiating from visitors’ entrance or exit, nor the transition zones (5.1; 5.4)
- The existence of an appropriate number of hand-washing stations accessible to all visitors regardless of age or height (5.11)
- The absence of automatic (or foot-operated) washing stations (6.6)
- The use of Personal Protective Equipment (PPE) (7.1; 7.2)

The human risk assessment procedure was carried out in five different phases.

Phase 1: Hazard identification: (1) Anthopozoonoses: in Table B in S2 Appendix is reported a list of the main zoonotic agents (both fungi and bacteria). (2) injuries: the visitor and staff involved in the AVI can suffer injuries caused by scratches and bites.
Phase 2: Hazard characterization (P): the results of this second phase are detailed in Table 3 and expressed in terms of probability (P).
Phase 3: Exposure Assessment: the results of the exposure assessment are detailed in Table 3 and expressed in terms of damage (D).
Phase 4: Risk characterization: the results of the risk characterization are detailed in Table 3 and expresses in terms of risk score (R) and risk categories (risk rating—RR) obtained with the existing control measures (Tables C-E in S2 Appendix).
Phase 5: Risk characterization: predicted with the additional control measure that could be implemented.

**Table 3:** List of relevant zoonosis in lemurs and other animals reported for animal-visitor interactions (detailed in Table B in S2 Appendix). Values of risk characterization for existing control measure and additional control measure to be implemented (phase 2–5). (P = probability; D = damage; R = risk score; RR = risk rating; OR = Oral route; DC = Direct contact; A = Aerosol; CM = Contact with infected material and ingestion; CF = Contact with body fluids; L = Low; M = Medium; H = High).

Hazard characterization–Exposure assessment			Risk characterization Phase 4 (Existing control measure)				Risk characterization Phase 5 (Additional control measure to be implemented)			
Exposure Condition/ Scenario	Hazard	Consequences	P	D	R	RR	P	D	R	RR
A / CF	*Mycobacterium tubercolosis*e *M*. *bovis*	Pulmonary tuberculosis/ extrapulmonary tuberculosis	1	3	3	L	1	3	3	L
OR	*Klebsiella pneumoniae*	Septicemia, abscess	2	2	4	M	1	2	2	L
OR	*Escherichia coli*	Mild/severe diarrhea; Haemolyticuraemic syndrome	2	3	6	M	1	3	3	L
OR	*Salmonella enterica*	Mild/severe diarrhea	2	2	4	M	1	2	2	L
OR	*Shigella* spp	Mild/severe diarrhea	2	2	4	M	1	2	2	L
OR	*Vibrio* cholera	Mild/severe diarrhea	2	2	4	M	1	2	2	L
OR	*Yersinia pseudotuberculosis*, *Y*. *enterocolitica*	Acute enterocolitis, diarrhea, septicemia	2	2	4	M	1	2	2	L
OR	*Campylobacter fetus* subsp *jejuni*	Bacteriaemia, Guillain-Barre syndrome	2	3	6	M	1	3	3	L
DC	*Leptospira* spp.	Kidney damage, liver failure meningitis, death	2	3	6	M	1	3	3	L
OR / A / DC	*Francisella tularensis*	High fever, chills, headache, focal ulcers, swollen lymph nodes	2	3	6	M	1	3	3	L
A / CM /CF	Methicillin resistant *S*. *aureus* (MRSA), Extended spectrum beta-lactamase (ESBL)	Skin infections, urinary tract infections (UTIs), intra-abdominal and respiratory infections	2	3	6	M	1	3	3	L
DC	Rabies lyssavirus	Cerebral dysfunction, death	1	3	3	L	1	3	3	L
DC / A	*Lymphocryptovirus*	Lymphadenopathy	1	2	2	L	1	2	2	L
OR	*Cryptosporidium* spp.	Mild/severe diarrhea	2	2	4	M	1	2	2	L
OR	*Giardia duodenalis*	Mild/severe diarrhea	2	2	4	M	1	2	2	L
OR	*Entamoeba* sp.	Stomach cramping, dysentery	2	1	2	L	1	1	1	L
OR	*Encephalitozoon cunicoli*, *E*.*intestinalis; E*. *bieneusi*	Diarrhea, Disseminated infection	2	2	4	M	1	2	2	L
DC	*Trichophyton mentagrophytes*	Reddish ring-shaped rash, that may be itchy/ eventually itchy	1	2	2	L	1	2	2	L
DC	Injuries	Bites and scratches	1	2	2	L	1	2	2	L

#### Visitor experience survey (Step D)

A total sample of 291 visitors answered questionnaires, N = 153 (53%) from the PostQ and N = 138 (47%) from the GenQ. Table 4 below summarizes demographic information and other independent variables collected from visitors who participated in “A tu per tu con i lemuri” activity with the PostQ, and from visitors interviewed with the GenQ nearby the zoo exit.

**Table 4:** Demographical information for PostQ and GenQ respondents.

Demographic	Category	PostQ respondents		GenQ respondents		X^2^	df	p-value
		Percentage	*n*	Percentage	*n*			
Sex	Female	52%	79	50%	69	0.072	1	.789
	Male	47%	72	49%	67			
	No Answer	1%	2	1%	2			
Age	14–18	7%	10	1%	2	9.412	5	.094
	19–25	12%	18	7%	10			
	26–34	17%	26	25%	35			
	35–54	55%	84	54%	75			
	55–64	5%	7	7%	10			
	65+	3%	4	3%	4			
	No Answer	3%	4	1%	2			
Education	Elementary school graduate	2%	3	1%	2	0.829	4	.935
	Middle school graduate	12%	19	10%	14			
	High school graduate	49%	75	49%	67			
	University degree	27%	41	30%	42			
	Higher degree / PhD	7%	11	8%	11			
	No Answer	3%	4	1%	2			
Education on nature/animals	Yes	12%	19	14%	20	0.252	1	.616
	No	86%	131	84%	116			
	No Answer	2%	3	1%	2			
Number of past visits	1	63%	97	64%	88	2.144	3	.543
	2–3 times	29%	45	30%	41			
	from 4 to 10	3%	4	4%	6			
	more than 10	3%	4	1%	1			
	No answer	2%	3	1%	2			
Annual ticket / Membership	Yes	4%	6	3%	4	0.227	1	.634
	No	95%	145	96%	132			
	No Answer	1%	2	1%	2			
Pet ownership	Have a pet	64%	98	66%	91	0.041	1	.839
	Not have a pet	33%	51	33%	45			
	No Answer	3%	4	1%	2			
Member of an environmental association	Yes	14%	21	22%	30	3.254	1	.071
	No	85%	130	77%	106			
	No Answer	1%	2	1%	2			

Among respondents of the GenQ (n = 138), only 20% (n = 27) of them joined the “A tu per tu con i lemuri” on the same day of the survey and the 46% (n = 64) of the interviewed participated at least one of any activity on that day. The most followed activities by GenQ respondents, among those proposed, were the bear talk (n = 28, 20%), the walk-in with lemurs (n = 27, 20%) and the penguin talk (n = 23, 17%). Similarly, among the PostQ respondents (n = 153), the most followed activities after the walk-in with lemurs were the bear talk (n = 38, 25%), and the penguin talk (n = 33, 22%). Therefore, it has been tested if any variables differed between visitors who joined the lemurs AVI and visitors who did not. The test did not find any statistical differences between the two groups for any variable tested.

No statistically significant difference was identified for any of the five statements investigated with the 5-point Likert Scale (S1 Table) between PostQ(n = 149) and GenQ (n = 135) visitors, nor between visitors who did the activity with lemurs (n = 176) and those who did not participate (n = 108). However, an independent-samples t-test indicated that statement B, “I don’t think I will take the time to learn more about animals”, scores were higher for weekend visitors (n = 173, mean = 2.08; SD = 1.17) than for weekday visitors (n = 111, mean = 1.84, SD = 0.89, t(282) = -1.787, *p* = .05).

There was no significant difference in the scoring given by the weekend visitors (n = 96, mean = 4.48; SD = 0.725) respect to the weekday visitors (n = 52, mean = 4.38; SD = 0.745) when asking them if they would suggest to friends to participate in the lemurs AVI (t = -.832, p = .407). The great majority of the PostQ answered that they absolutely would suggest to friends to participate (“absolutely probable”; n = 87, 59%). According with the Net Promoter Score (NPS), 59% (n = 87) of visitors could be considered as promoters, 28% (n = 42) as passive and 13% (n = 19) as detractors. According to the formula, the NPS resulted in 46%. However, the NPScalculated for the weekend visitors was higher than for the weekday visitors, resulting in 49% for the firsts and 40% for the second ones.

The pre-information experience was said to be satisfactory by 93% of the sample (n = 140), with a higher satisfaction level in the weekend visitors (99%, n = 96) compared to the weekday visitors (83%, n = 44). When asked why the pre-information was not satisfactory, the most frequent answer was that visitors had not received any information. As previously explained, during the weekday, the enclosure was opened by a keeper, and there was no provision of a guide to give information to visitors.

The majority of the attributes investigated with the Kano Model were a “Linear” requirement (Table 5), but “Direct contact with animals” and “Information about animals” were “Exciter” for 36% (n = 53) and 35% (n = 52) of the respondents, respectively. This means that for them the direct contact and information about the animals were unexpected attractive features, which provided high satisfaction. In Table 6 the CS coefficients for respondents’ satisfaction or dissatisfaction are presented.

**Table 5:** Attributes investigated with the Kano Model and their distribution within the categories.

Attribute	N (%)					
	Must have	Linear	Exciter	Indifferent	Reverse	Questionable
1. Direct contact	7 (5%)	21 (14%)	53 (36%)	34 (23%)	23 (16%)	10 (7%)
2. Information about animals	9 (6%)	44 (30%)	52 (35%)	36 (24%)	2 (1%)	6 (4%)
3. Information about conservation issues	18 (12%)	71 (48%)	27 (18%)	20 (13%)	4 (3%)	9 (6%)
4. Information about animal welfare	28 (19%)	87 (58%)	16 (11%)	12 (8%)	0 (0%)	6 (4%)
5. Presence of a guide	16 (11%)	83 (56%)	37 (25%)	8 (5%)	0 (0%)	3 (2%)

**Table 6:** Customer satisfaction and dissatisfaction coefficient (CS). The closer the satisfaction coefficient is to +1, the more the presence of the attribute influences respondents’ satisfaction. On the contrary, the closer the dissatisfaction coefficient is to -1, the more the absence of the attribute influences respondents’ dissatisfaction. If the CS is adjacent to 0, it means that the attributes have a low influence on visitor satisfaction or dissatisfaction.

Attribute	Satisfaction	Dissatisfaction
1. Direct contact	0.643478	-0.24348
2. Information about animals	0.680851	-0.37589
3. Information about conservation issues	0.720588	-0.65441
4. Information about animal welfare	0.72028	-0.8042
5. Presence of a guide	0.833333	-0.6875

### Overall ethical assessment (Steps E and F)

#### Ethical analysis (Step E)

Cells of the Ethical Matrix (EM, Tables 7 and S4) were populated to represent the ideal situation in which all the stakeholder’s moral demands, corresponding to the three *prima facie* ethical principles (well-being, autonomy, fairness), are respected during the AVI. From the comparison between the content of the EM cells and results obtained with the other steps, few non-conformities and potential conflicts were detected.

**Table 7:** Outline of the customized ethical matrix. Adapted from [24].

	WELL-BEING	AUTONOMY	FAIRNESS
ZOO ANIMALS PARTICIPATING IN THE AVI	Physiological and psychological welfare (LW)	Behavioral freedom (LA)	Intrinsic value (LF)
WILD ANIMALS AND THE ENVIRONMENT	Species and biodiversity conservation (WW)	Freedom from human intervention (WA)	Respect for the worth of every individual (WF)
VISITORS PARTICIPATING IN THE AVI	Physiological and psychological welfare (AW)	Self-determination (AA)	Fair treatment (AF)
VISITORS NOT PARTICIPATING IN THE AVI	Safety and psychological welfare (VW)	Self-determination (VA)	Fair treatment (VF)
KEEPERS INVOLVED IN THE AVI	Satisfactory and safety working conditions; professional realization (KW)	Professional freedom (KA)	Fair treatment (KF)
EDUCATORS INVOLVED IN THE AVI	Satisfactory and safety working conditions; professional realization (EW)	Professional freedom (EA)	Fair treatment (EF)
MANAGEMENT STAFF	Satisfactory working conditions; professional realization (MW)	Management freedom (MA)	Fair treatment (MF)
VETERINARY STAFF	Satisfactory working conditions; professional realization (VSW)	Professional freedom (VSA)	Fair treatment (VSF)
ZOO	Economic sustainability, support from society (ZW)	Mission fulfilment (ZA)	Adequate legislation and access to resources (ZF)

The lemur walk-in enclosure at Giardino Zoologico di Pistoia was opened in 2009, but only since 2013 the individuals involved in this study have moved in and started participating in the specific AVI. Veterinary records did not show any differences in the frequency of veterinary interventions between the five years before 2013 and the five years after (until this study was conducted). Results of this study did not indicate any adverse stressful effects on lemurs’ welfare, neither throughout the behavioural and physiological analysis, nor throughout the animal welfare risk assessment, therefore, it is possible to state that their well-being was respected (LW). Results indicate also that the enclosure allows a good degree of control on the environment also during the AVI. Visitor passage area within the enclosure represents only a third of the horizontal space of the lemur enclosure, and animals are not forced to be always visible, and they have free access to the internal area, which is never accessible to visitors (LA). Therefore, animals were shown to express most species-specific behaviours and use all the space available to them, respecting their well-being, autonomy and fairness (LW, LA, LF). The other statistically significant differences did not appear to be relevant in diminishing the welfare state of the animals. Moreover, since part of the zoo ticket is devoted to lemur’s in-situ conservation project, the five ring tailed lemurs at Giardino Zoologico di Pistoia appear to contribute to the conservation of their species (LF and WF).

The zoo entrance ticket includes every talk and activities offered within the park, including the AVI discussed here. Visitors are therefore respected in their freedom to participate in the activities and receive information about the animals without any additional cost and are free to choose between several alternative free talks and activities (AA, VA and VF). As results have shown, there is great participation (AA and VA, AF, and VF). Besides, the absence of an educator during the weekdays causes some concern about visitors’ well-being, autonomy and fairness, as they are more likely to be satisfied when receiving information about animals, animal welfare, and conservation issues during the AVI. Results of risk assessment showed low and medium risks for visitors’ health and safety. This means that, although there are signs and behavioural norms for visitors to follow within the enclosure, and staff monitors visitors’ compliance, there is some concern related to their well-being (AW). The implementation of additional control measures (e.g., periodic veterinary checks, as is routinely done at the Giardino Zoologico di Pistoia) reduces these physiological risks. On the other side, their psychological well-being, according to results of the surveys, was respected. The NPSwas 49%, and 59% of visitors answered that they absolutely would suggest to friends to participate. The CS coefficient confirmed, moreover, that direct contact and information about the animals had a high influence on respondents, which provided higher satisfaction to them (AW).

In this AVI, no concerns were identified regarding the management staff. The efforts of the management staff guarantee both animal welfare and the quality of the experience for visitors. It is therefore recognized that management staff has the necessary resources to train educators and keepers to meet the educational and entertainment needs of visitors, while ensuring the welfare of both visitors and animals involved (MW and MF). Both keepers and educators involved in AVI were satisfied with the working environment and their roles, thus respecting their wellbeing (KW and EW). They have adequate training in working with both animals and visitors during AVIs, and they are always available and ready to deal with any problems. Finally, as the results show, they contribute to fulfill the mission statement of the zoo, in terms of welfare, conservation and education (EF, KF and MF).

Veterinarians interact with all animals, including those involved in AVIs, and with most of the other stakeholders included in the EM. Veterinarians are essential with their work, as their activity have a crucial role in safeguarding the welfare of the animals and the safety of visitors. Therefore, their well-being and safety must be monitored. As in the case of the other human stakeholders, the risk assessment results showed low and medium risks of contamination (VSW). Moreover, veterinarians have discretionary power on how to monitor and protect animals, especially the ones involved in AVIs, have access to fair working conditions, and contribute to the quality of education and the implementation of biosecurity activities (VSA, and VSF). Therefore, in this AVI, no concern was identified with regard to veterinary staff.

Results also showed that Giardino Zoologico di Pistoia fulfills the goals concerning animal welfare and conservation education by offering the "A tu per tu con i lemuri" AVI (ZW, ZA and ZF). The zoo offers a wide range of educational and entertaining activities, where visitors are provided with multiple information. The participation in any of these activities, including the AVI and talks is free of charge. They are indeed included in the entrance ticket price, and part of it is donated to in situ conservation projects (ZF). Even if it was not possible to count the number of visitors participating in the interaction, the participation in the assessed AVI was high, both in the weekend and in the weekdays. Finally, results of the questionnaires showed that visitors were satisfied and willing to recommend the experience, thus contributing to the good reputation of the facility (ZW and ZA).

#### Final assessment (Step F)

The eleven entries checklist developed for the overall assessment recorded the following (Table 8):

**Table 8:** Final assessment checklist. Adapted from [24].

N.	Entry	YES	NO
1	The behavioural analysis did not identify any behavioural sign suggesting welfare issues (Step A).	x	
2	The analysis of physiological parameters (endocrine or others) did not identify any physiological sign suggestive of welfare problems (Step B).	x	
3	Only a negligible or low risk of welfare health was detected in the risk assessment analysis of physiological parameters (Step C1)	x	
4	No critical issues were detected when conducting an accurate ‘management and enclosure analysis’ within the welfare risk assessment (Step C1)	x	
5	A negligible or low risk was detected for the health/welfare of the people (visitors and staff) in the risk assessment analysis (Step C2)	x	
6	During the AVI, indications are given to increase awareness about wildlife conservation and animal welfare, and to promote sustainable behaviours among visitors (Step D)	x	
7	The visitor experience analysis detected a positive emotional impact (Step D).	x	
8	The visitor experience analysis detected a positive educational impact (Step D)	x	
9	The visitor experience survey detected a positive impact on the conservation mindedness and/or animal welfare awareness of the visitor (Step D)	x	
10	An ethical evaluation was done to highlight possible conflicts (Step E)	x	
11	If any ethical concern was identified with the AVI, the zoo staff is working toward a solution (Step E)	x	

## Discussion

Despite walk-in exhibits offering close animal encounters are common in modern zoos [1], their impact on animal welfare, health, and safety, as well as on visitors’ experience, education, and safety has been poorly researched. Bringing visitors into close proximity with animals is a form of interaction which needs to be carefully evaluated, even though it does not involve direct animal-visitor contact. Indeed, according to WAZA guidelines [6,7], it is of primary concern that the well-being of animals and the safety of visitors, as well as the educational and conservation impact of the proposed AVI are adequately monitored. AVIP stands as a key framework to fulfill these aims since it provides a holistic and interdisciplinary approach based on the integration of different assessment processes. In fact, it aims to evaluate animal physiological and behavioural welfare-related parameters, animal and human (both visitors and staff) health and safety risks, and educational and conservation outcomes. Results are then combined and compared with the content of an ethical matrix to carry out an ethical assessment which highlights key ethical concerns and informs whether the interaction is justifiable while suggesting potential practical solutions.

Currently, lemur walk-in enclosures are commonly present in zoos [26], and rigorous research to regularly assess this kind of AVIs is needed. The scientific studies assessing this kind of AVIs usually focus on few welfare parameters, or alternatively on visitors’ effects and exhibit design, or on educational contents, without fully exploring the undesirable outcomes [8]. AVIP allows an integrated assessment of lemur walk-in enclosure AVIs, evaluating the impact of these activities on both animals and visitors. The AVIP application described in this study allowed to assess for the first time different interconnected aspects during lemur-visitors interaction: animal welfare and safety, human welfare, safety and experience. Moreover, the ethical analysis, through the EM, allowed to analyze and reconstruct the framework of morally relevant interests considering the different stakeholders involved and the Overall Assessment provided a detailed result of the evaluation process suggesting improvements. Behavioural results, both at the group and individual level, indicated that the five ring-tailed lemurs were probably used to the presence of visitors during the AVI at the time of this study. The variations in grooming (self-grooming andgroomed by conspecific) detected in relation to the AVI (pre and post) both at the group and individual levels do not suggest a stereotypical overgrooming as no bald skin patches have been detected in any individual [49] and the percentage of time spent in them is low. Moreover, no differences have been found in the aggression levels. Thus, the variations in grooming do not indicate conciliatory contact [70]. This result agrees with Perry et al., (2011), and Hosey et al., (2016) that found that visitors do not affect lemurs’ behaviour or welfare, and visitors do not increase wounding rates [41,71]. Moreover, no other stereotypical behaviours were detected, although pacing has been evaluated as the common stereotypic behaviour in prosimians [54].While the H index did not detect any relevant significant difference associated with the AVI, it is interesting to note that the lemurs spent more time outside (in areas where also the visitors could stay) during the weekend morning interaction than during control sessions, suggesting that the animals did not feel threatened by the presence of visitors (which is corroborated also by the absence of an increase in scanning behaviour) [72], and they might also be interested in being outside where they could see the people inside the enclosure.

The physiological analysis further supported these results. Although the mean values of FGM concentration for each individual were lower in the weekdays, when there were fewer visitors in the zoo, no statistically significant difference has been found when more groups of visitors entered the enclosure. Overall, as the EM is suggesting, there were no significant differences suggesting an adverse visitor effect during the AVI (LW). However, due to logistical issues, it was possible to have control sessions only on weekday mornings. Still, it could have been interesting also to perform control sessions for each activity (morning/afternoon, weekend/weekday).

Most of the differences in behaviours are found between the two types of interactions and not between AVI and control sessions. However, differences that emerge between the two interactions (EEK and EE2) are not relevant to suggest a negative effect on the welfare of lemurs.The assessed AVI does not involve direct animal-visitor contact, although it allows visitors to closely observe the animals. Nonetheless, it also provides animals with the opportunity to express species-specific behaviours, to escape and avoid unpleasant stimuli. The enclosure design allows animals to have an always accessible retreat space and to avoid close proximity to humans, that might represent a source of stress, not only during AVIs but also for zoo animals in general [73]. Moreover, the resources present in the enclosure allow lemurs to express a diverse repertoire of behaviours, including grooming and affiliative behaviour, and facilitates the absence of intra-group aggression, satisfying their autonomy and wellbeing (LA and LW). These results suggest that at Giardino Zoologico di Pistoia lemurs are not negatively affected by the presence of the visitors inside the enclosure during the AVI (LW, LA). However, respect for visitors fairness (AF) can conflict with well-being and autonomy of the lemurs participating in AVI, if more people are interested in joining the activity (LW, LA).

Visitors were never observed performing behaviours that did not comply with the park’s rules. In rare situations where visitors showed negative behaviour towards lemurs (e.g., quick approach), the most common action of the animals was not responding or retreating. Furthermore, lemurs were never observed receiving food or voluntarily approaching visitors, even during the afternoon session on weekdays, when the supervision of keepers was not as close as during the weekend, and the number of visitors inside the enclosure at the same time was higher. Besides, even though all zoo staff was participative, it was not possible to have a count of visitors entering/exiting the enclosure for each activity and the time they spent watching the animals from outside the enclosure.

AVIP protocol foresees that the behavioural and physiological results obtained with the dedicated steps are the basis of the animal welfare risk assessment (phase C1). This phase allows for the calculation of the lemurs’ welfare risk, assesses the adequacy of the enclosure to maintain, during AVI activities, a high level of welfare for the animals involved, and examines the suitability of management procedures to prevent welfare risks to the animals. Therefore, the risks of spreading zoonoses and other health and safety problems of lemurs and visitors that may occur as a result of AVI were considered, analysed, and compared with the EM. In “A tu per tu con i lemuri” AVI, no animal welfare relevant consequences were detected. However, as a precautionary measure, the probability of disease spreading was defined as low and not negligible. In fact, possible future negative consequences on welfare cannot be excluded a priori, although periodical clinical examinations supported by laboratory tests for specific zoonotic agents are conducted. Through the development of three different scenarios, it was possible to classify the animal welfare risk as ‘low.’ However, in the third scenario, the WS reached 4, a threshold level of attention. This scenario considers the risks associated with zooanthroponosis. Therefore, an epidemiological investigation involving specific clinical observations and laboratory tests should accompany passive surveillance (permanent periodical veterinary control), leading to the detection of any new-onset zoonotic disease. However, the overall results of the Animal Welfare Assessment (steps A–C1) allow to conclude that the ideal situation of the well-being, autonomy and fairness regarding the interaction corresponded with the actual situation, with no need to discontinue the AVI.

AVIs must convey educational and conservation messages, since combining visitor-animal interactions with an educational experience may enhance visitor learning [2,3,29]. The presence of a guide and the information about conservation issues and animal welfare have been shown to strongly influence visitors’ satisfaction following results from the surveys, respecting their wellbeing, autonomy and fairness (AW, AA, AF). From the comparison of the EM and the Human Outcome Assessment results appears that visitors were mainly satisfied with the information given before the beginning of the activity. In general, people visiting the Giardino Zoologico di Pistoia chose to attend these activities, although many of them took place late in the afternoon. As questionnaires were administered in the afternoon (around 5 p.m.), some talks were not performed yet, and visitors’ participation to some of them was not recorded.

However, despite general satisfaction of visitors, the absence of a guide and the relative information provided during the weekdays, influenced respondents’ dissatisfaction, as also shown by the CS coefficients. The absence of information given to visitors did not affect animal welfare but created a concern for visitors’ well-being, autonomy and fairness (AW, AA, and AF). Due to the educational role these activities offer to visitors, it is important to give visitors information not only about how to behave during the AVI but also about the individual animals, their welfare and conservation.

Direct contact with the animals during the interaction is a critical issue in all AVIs as it could be linked to the risk of contamination. Therefore, the AVIP Human Outcome Assessment includes a specific risk assessment to carefully evaluate the risks related to human health during the AVI. In the assessed AVI, low and medium risks for visitor health were detected, creating a risk for their well-being (AW). The additional measures that would bring the risk from medium to low are mainly related to items 5.11 and 6.6 (Table A in S2 Appendix). Consequently, a service access point differentiating visitor entrance and exit, or transition zones, and the use of PPE are measures that should be implemented to enhance visitor safety. However, implementing the use of PPE could have a negative psychological impact on the animals and on visitor experience because the animal could be perceived as dangerous. Moreover, the presence of a safe area and the possibility of storing personal items near the enclosure should be considered not only for the visitors’ safety, but also for their experience, and for respecting their well-being (AW). In this analysis, SARS-CoV-2 was not considered as this study was conducted before the emergency of the CoViD-19 epidemic. At present, however, it is difficult to adequately perform the hazard characterization phase as scientific studies have not yet provided robust scientific evidence regarding the susceptibility of these animals to SARS-CoV-2.

AVIP must always be customized to the specific AVI. The specific application of AVIP to the lemur walk-in at Giardino Zoologico di Pistoia, compared to past AVIP applications (e.g., giraffe feeding [24,42], turtle experience [not published], etc.), had some limitations. First, due to the design of the AVI proposed to the Giardino Zoologico di Pistoia’s visitors, it was not possible to perform a pre-questionnaire before the beginning of AVI, and a matched post-questionnaire to the same visitors, to test the educational impact of the activity on visitors (as in [24,74]), nor to perform a long-term follow-up data collection (as in [75,76]). The inclusion of this assessment is important as AVIs must convey educational contents. A second factor was the assessment of the visitor effect. The control situation recorded in this study is not sufficient to extrapolate the visitors effect from the behavior of the animals, also because visitors, although not present within the enclosure, could see lemurs from different positions from outside the enclosure and consequently, lemurs could constantly see visitors [16]. Therefore, it is not possible to exclude that there have been reciprocal influences between the behavior of animals and that of visitors. As recommended by Goodenough et al., (2019) the weather was considered, as they demonstrated that it is a factor exerting a strong effect on the behavior of lemurs [28].

As a result of the overall assessment process, AVIP highlights strengths and weaknesses of the AVI under evaluation and proposes management decisions to address concerns depending on the outcome. Regarding the specific “A tu per tu con i lemuri” AVI, the main suggestions for improvement arising from the AVIP assessment were i) to implement the presence of a guide also during weekdays to assist visitors in obtaining information, ii) the placement of lockers to store personal items before AVI, and iii) periodical meetings for the staff involved in AVI (keepers and educators) to recall them the correct rules to be followed in order to continue to safeguard the health and welfare of animals and visitors. However, for an exhaustive assessment it would be also advisable to assess the specific educational impact of the walk-in enclosure on both weekend and weekday visitors. Anyway, from an ethical point of view, and from the comparison between the ideal situation described in the EM and the results obtained with the A-D steps, it is possible to conclude that the zoo addressed the highlighted concerns notwithstanding the complex framework of the AVIs. In the final checklist no negative answers were recorded.

Although it would be interesting to do a multicenter study to examine the different AVIs involving lemurs in different zoos, the protocol is not meant to compare different interactions, as differences in animals’ age, group composition, characteristic of AVI, etc., as well as subjective variability of individuals, would not allow relevant comparisons between different facilities. Nonetheless, even if a more detailed evaluation of every single aspect involved in the AVI (e.g., behavioural assessment, physiological assessment, educational impact, etc.,) could eventually offer more in-depth and comprehensive inputs, AVIP promotes a holistic framework which satisfies WAZA recommendations and the One Health–One Welfare approach. Its outcome could help zoos willing to perform an AVI self-assessment through AVIP, as the Giardino Zoologico di Pistoia did, to develop targeted interventions to optimize both animal welfare,health and visitors experience, and safety during the specific AVI under assessment.

## Conclusions

Given the increasing demand for AVI activities, and their supposed educational and recreational value, a multidisciplinary tool is crucial to evaluate their impact both on animals, visitors and the staff involved. AVIP is designed to support each facility in the self—assessment of its interaction activities. The aim of AVIP is to monitor all key aspects of a specific interaction, and to ensure that welfare of the animals, their safety and safety of visitors are carefully considered. AVIP results can support the work and commitment of zoos to both animal welfare and conservation education, while assisting in improving their management decisions and ensuring a transparent evaluation of their activities.

Result of AVIP application which are presented here are related to a specific AVI involving five lemurs at Giardino Zoologico di Pistoia and the specific circumstances at the time in which the study was performed. The results highlighted that there is no need to discontinue the assessed AVI as no concerns for lemurs, visitors, and staff were found, and ethical concerns were well addressed. This application shows that AVIP can be useful also in assessing walk-in enclosures with *Lemur catta* in zoos, confirming its potential to address WAZA recommendations for an overall evaluation of animal-visitor interactions.

## Supporting information

S1 Fig Diagram of the observational schedule. (DOCX)

S2 Fig Diagram of the ring-tailed lemur enclosure (“Voliera dei lemuri”) at Giardino Zoologico di Pistoia. (DOCX)

S1 Table Relevant adaptations in the materials and methods applied for the AVIP A-E steps. (DOCX)

S2 Table Ring-tailed lemurs ethogram. Working ethogram used in the study (adapted from [45–54]. Behaviors marked with “§” represent *events*.
(DOCX)

S3 Table Medians, interquartile range of the behaviours which resulted significantly different among sessions and Mann-Whitney U-test results performed at the individual level. Only behaviours which significantly differed or showed a tendency to differ are reported.
(DOCX)

S4 Table Customized ethical matrix. Adapted from [24].
(DOCX)

S1 Appendix Animal welfare risk assessment. Tables (Tables A–E) used to perform the animal welfare risk assessment.
(DOCX)

S2 Appendix Human risk assessment. Tables (Tables A–F) used to perform the human risk assessment.
(DOCX)
