# Supplementary figures and images for: The Animal-Visitor Interaction Protocol (AVIP) for the assessment of Lemur catta walk-in enclosure in zoos
Source: PLoS One. 2022 Jul 28;17(7):e0271409. doi: 10.1371/journal.pone.0271409 (PMC9333233; doi:10.1371/journal.pone.0271409)

**S1 Fig. Diagram of the observational schedule**


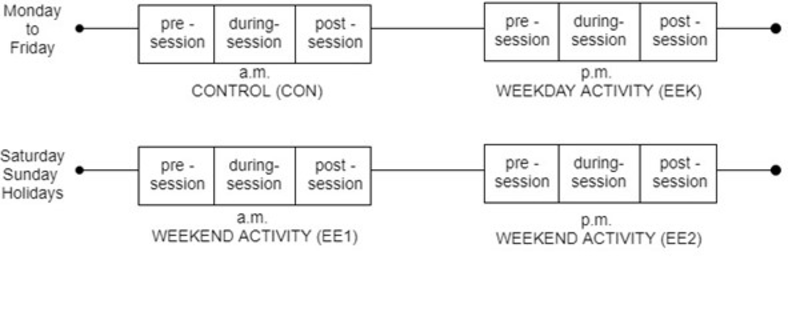

Supplement: S1 Fig — (DOCX) [file pone.0271409.s001.docx]
