# Supplementary material for: The Animal-Visitor Interaction Protocol (AVIP) for the assessment of Lemur catta walk-in enclosure in zoos
Source: PLoS One. 2022 Jul 28;17(7):e0271409. doi: 10.1371/journal.pone.0271409 (PMC9333233; doi:10.1371/journal.pone.0271409)

**S2 Fig**. **Diagram and photo of the ring-tailed lemur enclosure (“Voliera dei lemuri”) at Giardino Zoologico di Pistoia.**

Seven different areas have been identified (1,2,3,4,5,6, RI). View of areas 6, 4, and 2 from the entrance door.


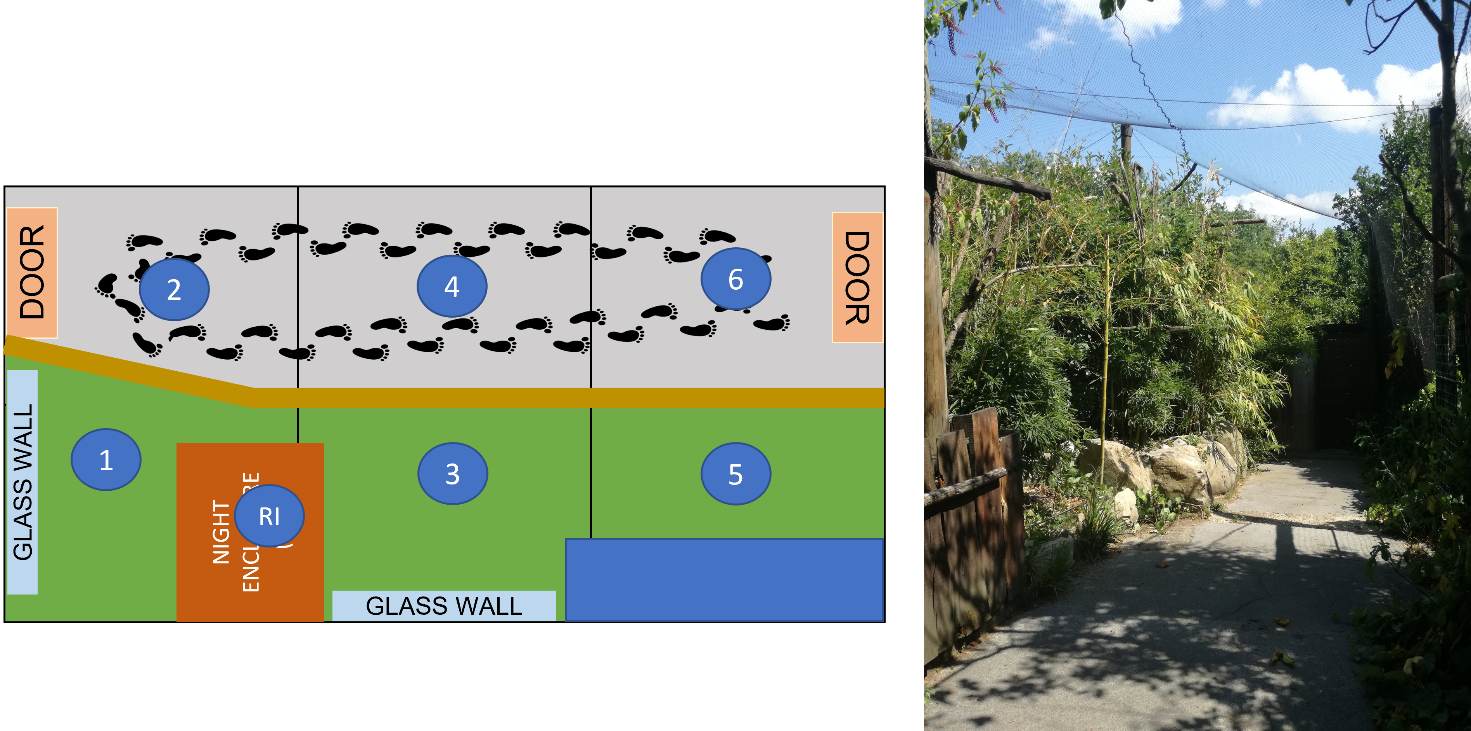

Supplement: S2 Fig — (DOCX) [file pone.0271409.s002.docx]
