# Supplementary material for: The Animal-Visitor Interaction Protocol (AVIP) for the assessment of Lemur catta walk-in enclosure in zoos
Source: PLoS One. 2022 Jul 28;17(7):e0271409. doi: 10.1371/journal.pone.0271409 (PMC9333233; doi:10.1371/journal.pone.0271409)
Supplement: S1 Table — (DOCX) [file pone.0271409.s003.docx]

**S1 Table. Relevant adaptations in the materials and methods applied for the AVIP A-E steps**

| **Step** | | **Component** | **Phase** | **Description** | **References** | |  |
| --- | --- | --- | --- | --- | --- | --- | --- |
| **A** | Behavioural Observation | Observational schedule |  | For 26 interaction episodes (8 afternoon interaction episodes of the weekday - EEK; 10 morning interaction episodes of the weekend - EE1; 8 afternoon interaction episodes of the weekend - EE2), recordings took place:  • For 25 min (five minutes per animal, randomized order) 30 min before interaction began (pre-session).  • During the interaction (one minute per animal, for two interactions; same order as in the corresponding pre-session, during-session).  • For 25 min after the interaction (five minutes per animal, same order as in the corresponding pre-session, beginning 20–30 min after interaction ended, post-session).  For 10 control episodes (similar environmental conditions as the morning interaction episodes of the weekend - CON), recordings were done with analogous time scheduling as for the interaction episodes (pre-, during-, post-sessions). | S1 Figure | |  |
|  |  | Behavioural data gathering methods |  | The *continuous focal animal sampling* method was used to record and collect the durations of individual behavioural patterns [43, 44] performed by the lemurs. The ethogram used for the observational study was prepared based on preliminary observation and previous studies on *Lemur catta* and related species [45-54].. | S2 Table | |  |
|  |  | Other collected data |  | During each interaction episode the temperature (°C) inside the enclosure was collected. Moreover, during the behavioural recording, the position of each lemur within the enclosure (S2 Figure) were recorded. | S2 Figure | |  |
| **B** | Physiological measures | Data gathering methods |  | Individually identifiable faecal samples were collected every time the researcher saw the animal defecating. Faecal samples were generally collected within a few minutes from the outside enclosure. Samples were collected in small plastic bags, sealed, labelled with animal ID, date and time, and then stored at -20°C until further analysis. |  | |  |
|  |  | Sample analysis |  | An aliquot of each sample was subjected to an organic phase extraction using ethanol. Extraction and determination of FGM were carried out as previously reported by [62]. |  | | |
| **C1** | Animal Welfare Risk Assessment | Goal |  | Identify any risk for the animals’ welfare, following the methodology provided by the EFSA guidelines [57], which allows calculating a Welfare Score. For the Welfare Score estimation, a semi-quantitative method was used. |  | | |
|  |  | Problem formulation | Risk question | What are the consequences on welfare if a management model that provides for a human-animal contact is introduced in comparison to a management model where that contact is absent? |  | | |
|  |  |  | Target population | A group of five ring-tailed lemurs (*Lemur catta*) |  | | |
|  |  |  | Identification of factors of animal welfare concern | The interaction itself (because, even if there is no direct contact with animals, visitors entering the enclosure could cause unpleasant subjective experiences, injuries or diseases due to an incorrect approach to animals); the enclosure design where the activity occurs; management factors, identified with the ”Management Checklist: Staff Actions and Procedures”. | Table A in S1 Appendix | | |
|  |  |  | Exposure scenario | The exposure scenarios that were identified assumed that the management model with AVIs could negatively affect animal welfare. |  | | |
|  |  |  | Animal welfare consequences and their measurement | The consequences could be behavioural alterations following the worsening of subjective experiences (scenario 1), traumatic injuries (scenario 2) and infections (scenario 3). |  | | |
|  |  |  | Conceptual models | In the management model with the interactive activities, the conceptual model includes the three different scenarios. The first two scenarios recognize a common factor. We defined that an improper approach is a factor that can determine behavioural alterations that could indicate the perception of negative subjective animal experiences (scenario 1), and that could also provoke injuries (scenario 2). Scenario 3 defines as exposure factor a contact with a zoonotic agent that can cause infectious diseases. |  | | |
|  |  | Welfare risk assessment | Exposure assessment | The factors described in the three scenarios were assessed using a semi-quantitative method to estimate the frequency of exposure. | Table B in S1 Appendix | | |
|  |  |  | Consequence characterization | Three animal-based indicators were created to quantify the severity of the consequences identified in the different scenarios. | Table C, D, E in S1 Appendix | | |
|  |  |  | Risk characterization | The Welfare Score (WS) = FE (Frequency of exposure to the factor in a specific scenario) × MA (Magnitude as a product of the severity of the consequences for their duration in a specific scenario) × FC (Frequency of consequences in a specific scenario). |  | | |
| C2 | Human Risk Assessment | Goal |  | The visitor safety risk assessment was carried out referring to and adapting the documents of the Department for Environment, Food, and Rural Affairs [58]. This process aims to highlight possible risks to the safety and health of visitors, to identify measures to eliminate or reduce dangerous exposure, and to find preventive and protective actions to ensure the protection of people. |  | | |
|  |  | "Management Checklist": Preventive and Protective Measures" |  | Analyse both existing prevention and protection measures and other actions that can be implemented. During its compilation, the researcher recorded a yes response whenever the action included in the checklist was seen or visual evidence of structural requirements or documentary evidence was found. | Table A in S2 Appendix | |  |
|  |  | Hazard identification | Phase 1 | Based on scientific reports of diseases in the animal species involved in the AVI, an exhaustive list of potential biological and physical hazards was created. | Table B in S2 Appendix | |  |
|  |  | Hazard characterization | Phase 2 | The probability of people being exposed to a specific hazard was calculated by creating four different categories and assigning to each of them a numerical value on an ordered scale from 1 to 4 and taking into account existing preventive measures. | Table C in S2 Appendix | |  |
|  |  | Exposure assessment | Phase 3 | The exposure assessment focuses on the extent or magnitude of the damage. Also in this case, four different categories were established to represent the extent of the damage, taking into account the existing protective measures. | Table D in S2 Appendix | |  |
|  |  | Risk Characterization | Phase 4 | Based on the outcome of phase 2 and phase 3, the risk was calculated. The resulting risk categories were then defined by checking whether certain mitigation activities should be implemented and their urgency. | Table E and Table F in S2 Appendix | |  |
|  |  | Risk mitigation | Phase 5 | This is a reiteration of phases 2–4 after an analysis of the additional preventive and protective actions in the case they must be implemented (Ki) to mitigate the risk. |  | | |
| **D** | Visitor Experience Survey | Goal |  | Surveys were developed to investigate visitors’ perception of the experience, self-reported changes in visitors’ attitude towards animals and conservation issues. |  | |  |
|  |  | Survey design |  | “Preview Visitor Experience Analysis Checklist on Educational Aspects” allowed the development of two different questionnaires structured as follow:  Post-interaction questionnaire (PostQ): 13 items divided into six sections;  General questionnaire (GenQ): 10 items divided into four sections.  Both questionnaires consisted of: demographical questions (age, sex, etc); multiple-choice questions to investigate the activities (e.g., talks, close encounters with animals, etc.,) done by the visitors during their zoo day; 5-point Likert scales (where 1=strongly disagree and 5= strongly agree) to investigate five statements (A= I am going to talk about animals to my friends; B= I do not think I will take the time to learn more about animals; C= I intend to support zoo conservation projects; D= I intend to support conservation projects of other organizations; E = I have no intention of changing any of my daily activities to promote the environment). The Kano Model [68,69] was used for a more in-depth analysis of visitor satisfaction, selecting five different attributes of the proposed interaction: 1) Direct contact with the animal; 2) Information about the specimens involved in the interaction (age, gender, biographical info, etc.); 3) Information about convenient behaviours for the conservation of the species involved; 4) Instructions given on how visitors should behave in order not to compromise the welfare of the involved animals; 5) Presence of a guide during the activity. The Net Promoter Score was applied to measures the willingness of the activity participants to recommend the lemurs AVI to friends on a 5-point scale (where 1 = “absolutely not probable” and 5 = “absolutely probable”). |  | |  |
|  |  | Data gathering methods |  | Participation in the survey was voluntary and anonymous. School groups, staff, zoo volunteers, and persons under 14 years of age were excluded from the study. The researcher gently stopped the visitors, explained the aims of the study, and provided them with the questionnaire, a pen, and sheet support. PostQ questionnaires were administered to visitors who were exiting from the Lemurs’ enclosure at the end of the activity. GenQ questionnaires were administered in the afternoon (around 5 p.m.) to visitors who were exiting the zoo gates at the end of their visit, using systematic sampling to select them (every two units: Single visitor or group). |  |  |  |
| **E** | Ethical Analysis | Ethical Matrix Methods |  | The identified interest groups who have an ethical standing in the AVI were: zoo lemurs (L); wild lemurs and environment (W); visitors participating in the AVI (A); visitors not participating in the AVI (V); Keepers (K); Educators (E); management staff (M); veterinary staff (VS); zoo (Z).  By a top-down approach, the Ethical Matrix [60] was populated by the researchers considering the ideal situation about the AVI, in which the need and values for all the relevant stakeholders that have been identified were respected. | S4 Table | |  |
|  |  | Ethical assessment |  | For the ethical assessment of the AVI under investigation, the results obtained in the previous steps were compared with each cell of the customized Ethical Matrix to identify possible ethical concerns, both in terms of potential conflicts between different cells and concerns emerging from non-conformities. |  | | |
