# Supplementary material for: The Animal-Visitor Interaction Protocol (AVIP) for the assessment of Lemur catta walk-in enclosure in zoos
Source: PLoS One. 2022 Jul 28;17(7):e0271409. doi: 10.1371/journal.pone.0271409 (PMC9333233; doi:10.1371/journal.pone.0271409)
Supplement: S2 Table — Working ethogram used in the study (adapted from [45–54]. Behaviors marked with “§” represent events. (DOCX) [file pone.0271409.s004.docx]

**S2 Table: Ring-tailed lemurs ethogram.** Working ethogram used in the study (adapted from [45-54]. Behaviors marked with “§” represent *events*.

| **Behavioural category** | **Behaviour** | **Description** |
| --- | --- | --- |
| Postures | Standing | The animal is in an upright position and stationary, with all or only two legs on the ground and legs extended, supporting the body. While standing, the animals may look around their surroundings. |
|  | Sitting | The animal is stationary. The animal sits, with forelegs extended, hind legs flexed, with head up and eyes open. While sitting, the animals may look around their surroundings. |
|  | Sleeping | The animal lies down, stationary, with the head down. The eyes may be closed. |
|  | Hanging | The animals suspend themselves from branches, grasped with both their feet (bipedal hanging) or with their feet and one hand, or with the hands (uni- or bimanually hanging). The body is fully downward, and the animals remain motionless for short periods. This posture is shown often while the animal is feeding, grooming or playing. |
|  | Sunbathing | Thermoregulation activity. During the “sunbathing” behaviour, the animal exposes its body to the solar heat in an upright sitting position, with its arms extended laterally or resting on its legs. The head is tilted back, and the eyes are frequently closed. |
| Maintenance | Scanning | Animals show attention (i.e., appears to direct its gaze) toward a specific stimulus by interrupting what there are doing, for at least 8 seconds. The animal observes the surrounding environment while it is sitting or standing on the ground/horizontal supports/tree branches. The back is held vertically or variously inclined. Arts are flexed or bent. |
|  | Sniffing § | The animal smells anything that surrounds him (air, conspecifics, branches, soil, visitors, bags or backpacks), by inhaling air through the nose in a fast repetitive way. |
|  | Urinate/Defecate § | The animal releases urine or defecates on the ground without a specific pose, but with a lowered tail |
|  | Self-grooming | The animal cleans itself by licking, scratching, biting, or chewing the fur on its body also using the toothcomb. Licking is often alternated with combing. The primary parts self-groomed are limbs, torsos, sides, genital regions, and tails. They also lick their digits and scratch their heads, arms, torso. |
|  | Yawn § | The animal opens its mouth widely while inhaling, then closes the mouth while exhaling deeply. |
|  | Scratching § | The animal scratches its body using its feet or hands. |
|  | Licking-object | The animal tongue protrudes from the mouth and strokes non-food objects or the soil. |
| Terrestrial locomotion | Walking | Forward locomotion at a slow gait, quadrupedal stance and the tail is held aloft. |
|  | Running | Terrestrial forward locomotion in a rapid gait, which is faster than walking. Running is characterized by a bounding motion. While running, animals thrust their bodies off of the ground and extend their legs. The contact with the ground is regained first by the hands and the gait is completed when their feet landed just behind the position of the hands. |
|  | Jumping § | The animal leaps from one point to another, either vertically or horizontally. By pushing off with all four legs and keeping its torso horizontal, the lemur moves forward before dropping back to the ground, landing on all four limbs. In jumping from the ground to a branch or tree trunk, initially it contacts the target substrate with its hands, immediately before bringing its feet into contact with the support. The body is oriented vertically during this type of jump. |
| Feeding | Natural | The animal actively uncovers or searches for a naturally-occurring food item, and ingests vegetation, such as leaves, grasses, buds, flowers, and acorns foraged along the 'natural' part of the enclosure or trees, by means chewing with teeth and swallowing. As they chew the animal hold their heads at a vertical or near-vertical orientation, apparently to keep the food between cheek teeth. The animals either sit or stand on all four legs while eating. |
|  | Provisioned | The animal ingests the food of the zoo diet by means chewing with teeth and swallowing. The pieces of food are completely ingested, or they bite only a portion. As they chew, the animals hold their heads at a vertical or near-vertical orientation, apparently to keep the food between cheek teeth. The animals either sit or stand on all four legs while eating. |
|  | Drinking § | The animal ingests water |
| Marking | Scent Marking § | The animal uses glands (such as those located under the tail, arms, wrist) to mark the surfaces. Both females and males scent mark. Females scent marks with the anogenital region only, whereas males mark with the anogenital region (Anogenital marking), with the spur glands rubbed against branches, trees or objects (spur marking), or the spur glands rubbed against the tail which is then carried over the animal's body and waved (wave tail). Moreover, the males can scent mark rubbing the spur glands present on the ventral surface of the wrists or those present over the arms along the tail that is held between the legs and kept erect in front of the back (Anoint tail). |
| Affiliative social behaviours | Huddling | The animal is at rest, lying, sitting or sleeping with body in contact with other individuals (at least one other individual). |
|  | Grooming conspecific | A lemur grooms another one by licking, scratching, biting, or chewing the fur on the other body also using the toothcomb. |
|  | Groomed by conspecific | One lemur is groomed by another one who licks, scratches, bites, or chews his fur. |
|  | Mutual grooming | Two animals groom each other at the same time. The two individuals are usually placed one in front of the other and they mutually lick, scratch, bite or chew the fur on the other body, also using the toothcomb. |
|  | Group movement | The animals move as a group. Any group member can initiate the progression by moving in a particular direction, stimulating the others to follow as a loose aggregation. |
| Agonistic Social Behaviour | Aggressive § | Behaviours that clearly have the potential to hurt the opponent. The ears could be flattened against the head. It includes:  Bite: The individual orally seizes or slashes another individual by sinking the canines into the rival and then tearing them apart violently. Charge: The individual quickly moves towards another individual so that the other animal is displaced from where he is, but then he stops at the point occupied first by the latter; there is no chase, and it is not followed by play. An animal moves with straight and rigid legs, keeps its head and tail high and straight and continues to stare at the animal to which it is heading. Cuff: to give one or two slaps, alternating the hands, on the *muzzle* of another individual, scratching it with the nails or tearing off a tuft of hair (Full cuff). The animal, remaining still where it is, could also raise its arm and makes a rapid forward movement of the shoulder, towards the animal to be hit, but, in reality, it does not even touch it (Sham Cuff). Chase: an animal chases another individual, chasing him on the ground or climbing up to reach him. Group chase: two or more animals chase another one, chasing it on the ground or on the branches, and trying to approach it from several directions. Stare: an individual keeps his/her widened eyes fixed on the individual to be threatened, with a rigid body posture. Jump fight: two individuals face each other on the ground, one in front of the other, in a competition of jumps on two legs, holding their arms up and wide, they jump around each other trying, in the descent, to hit, scratch and bite the opponent. Grimace: the animal opens the mouth, retracts the lips, and shows the teeth. |
|  | Submission § | Actions used to evade or appease aggression and include different behaviours. Control: a chased animal, while escaping stops and turns to look at his pursuer, with an alert position - the ears straight forward and the eyes fixed on the other individual. Glance: the animal that is threateningly fixed (Stare), lowers or turns its eyes away by looking at other points or turns its head. Jump away: the individual who has been charged by a dominant animal jumps away from the point where he was, but then stops and there is no real escape. Run away: the individual who is chased, or has been charged, runs away running or jumping on other branches, tries to get away from his pursuer. Escape at the sight of: an animal, at the sight of another one, moves away from where it is. Being displaced: move away immediately after being fixed or approached by a conspecific. |
| Vocalization | Vocalize § | The animal opens the mouth and emits sounds or calls. The vocalization could be of different types (e.g., Territorial calls, Mew, Moan, Howl, Purr, Gulp, Chirp). |
| Play | Play | During the play behaviour, a lemur interacts with a group member without the intention to harm. Typically includes non-agonistic fighting and chasing. The play behaviour includes tail-play and a relaxed open mouth. |
| Stereotypic | Stereotypical behaviours | Pace: Repetitive locomotion in a relatively invariant pattern, such as back and forth along the same route. Can include walking and running. Movement seems to have no apparent goal or function. Must be performed at least two times in succession before qualifying as stereotypic. Somersault: The animal repeatedly tosses its head in a circular motion. This behaviour does not occur during play or agonistic interactions, but can occur while performing another stereotypic behaviour, such as pacing. Overgroom: The animal cleans itself or another individual excessively. May result in bald patches of fur. Self-Injurious: The animal uses teeth, claws, or nails to cause harm to itself, such as self-biting or chewing. |
| Not Visible | Not visible | The animal spends time “out of sight”, and it is not possible to see what the individual is doing. In particular, the lemur is behind trees and bushes or in the internal area. |
| Others | Other behaviour | The animal is visible but is engaged in any behaviour that does not fit into one of the descriptions provided above. |
