# Supplementary material for: The Animal-Visitor Interaction Protocol (AVIP) for the assessment of Lemur catta walk-in enclosure in zoos
Source: PLoS One. 2022 Jul 28;17(7):e0271409. doi: 10.1371/journal.pone.0271409 (PMC9333233; doi:10.1371/journal.pone.0271409)
Supplement: S3 Table — Only behaviours which significantly differed or showed a tendency to differ are reported. (DOCX) [file pone.0271409.s005.docx]

**S3 Table**. Medians, interquartile range of the behaviours which resulted significantly different among sessions and Mann-Whitney U-test results performed at the individual level. Only behaviours which significantly differed or showed a tendency to differ are reported.

| Subject | Behaviour | Session | Median | IQR | Mann-Whitney U-test | P-value |
| --- | --- | --- | --- | --- | --- | --- |
| Sakalava | Standing | EEK - post | 0.10 | 0.25 | 13 | 0.05 |
|  |  | EE2 - post | 0 | 0.03 |  |  |
|  | Walking | EEK - post | 0.15 | 0.32 | 13 | 0.05 |
|  |  | EE2 - post | 0.01 | 0.11 |  |  |
|  | Not Visible (on the total time) | EEK - post | 0.25 | 0.51 | 13.5 | 0.05 |
|  |  | EE2 - post | 0 | 0.13 |  |  |
| Ankarana | Self-grooming | CON - pre | 0.07 | 0.08 | 23 | 0.04 |
|  |  | EE1 - pre | 0.17 | 0.36 |  |  |
| Mandrare | Self-grooming | EEK - pre | 0.12 | 0.20 | 23 | 0.04 |
|  |  | EE2 - pre | 0 | 0 |  |  |
