# Supplementary material for: The Animal-Visitor Interaction Protocol (AVIP) for the assessment of Lemur catta walk-in enclosure in zoos
Source: PLoS One. 2022 Jul 28;17(7):e0271409. doi: 10.1371/journal.pone.0271409 (PMC9333233; doi:10.1371/journal.pone.0271409)
Supplement: S4 Table — Adapted from [24]. (DOCX) [file pone.0271409.s006.docx]

**S4 Table. Customized Ethical Matrix.** Adapted from [24]

| **Stakeholders** | **Well-being** | **Autonomy** | **Fairness** |
| --- | --- | --- | --- |
| ZOO ANIMALS PARTICIPATING IN THE AVI | Improving animal health and avoiding risks to animal welfare; ability to express normal patterns of behaviour; being kept in an appropriate space, with an adequate number of individuals; living a life worth living for a wild animal in human care. (LW) | Behavioural freedom: ability to choose not to interact with visitors; being able to have a degree of control on the environment. (LA) | Being considered with respect as “ambassador animals” contributing to the conservation of the species in the wild. Same opportunities for positive welfare outcomes as other animals that are not involved. (LF) |
| WILD ANIMALS AND THE ENVIRONMENT | To have an adequate environment in which to live, without natural and/or human threats that endanger their conservation; living a life worth living in the natural habitat. (WW) | Having the freedom to choose where to live and to reproduce; having the availability of sufficient resources. (WA) | Improving animal health and avoiding risks to animal welfare; ability to express normal patterns of behaviour; being kept in an appropriate space, with an adequate number of individuals; living a life worth living for a wild animal in human care. (WF) |
| VISITORS PARTICIPATING IN THE AVI | To see exotic and wild animals and learning information and be educated about them. Safety and secure environment and amusement. (AW) | To see the animals; have access to information on animals, on environments and on conservation; possibility to do educational and entertainment activities; take part in projects of conservation of nature. (AA) | Affordability; be physically and emotionally close to the nature and animals; to spend pleasant days and to do emotional and educational activities. (AF) |
| VISITORS NOT PARTICIPATING IN THE AVI | Learning information and being educated about wild animals if interested in; safe and secure environment; to support the zoo mission statement and activities. (VW) | Having access to all the information about the activities; having the freedom to choose whether to participate in the activity or not; having the opportunities to see the animals; having access to the information on animals, conservation and environments; being free to take part in conservation projects. (VA) | Equal educational opportunities and equal opportunities of accessing the natural resources; equal right to being physically and emotionally close to nature and animals; equal right to benefit from the mission statement of the zoo (in terms of welfare, conservation, and education). (VF) |
| KEEPERS INVOLVED IN THE AVI | Having an economically rewarding and comfortable job; working in a safe and secure environment, also during the interactions. (KW) | Being able to choose the tasks that best reflect personal skills and values; being able to be part of the management strategies to promote the well-being of the animals involved in the interactions; being able to be part of the management strategies to promote the conservation and education activities related to interactions; being able to work independently (in terms of space, instruments, skills, and education) and respecting the law; to be always updated on the new research about animal welfare, conservation, education and the current legislation. Being able to participate in scientific projects and for the protection of nature. (KA) | Respect for their role; equal right to professional practice; equal access to funds to develop and grow; to be able to have a clear and adequate legislation that protects at the work-place; contributing to the fulfilling of the mission statement of the zoo, in terms of welfare, conservation and education. (KF) |
| EDUCATORS INVOLVED IN THE AVI | Having an economically rewarding and comfortable job; working in a safe and secure environment, also during the interactions. (EW) | Being able to choose the tasks that best reflect personal skills and values; being able to be part of the management strategies to promote the well-being of the animals involved in the interactions; being able to be part of the management strategies to promote the conservation and education activities related to interactions; being able to work independently (in terms of space, instruments, skills, and education) and respecting the law; to be always updated on the new research about animal welfare, conservation, education and the current legislation. Being able to participate in scientific projects and for the protection of nature. (EA) | Respect for their role; equal right to professional practice; equal access to funds to develop and grow; to be able to have a clear and adequate legislation that protects at the work-place; contributing to the fulfilling of the mission statement of the zoo, in terms of welfare, conservation and education. (EF) |
| MANAGEMENT STAFF | Having an economically rewarding, comfortable job; working in a safe and secure environment; having the professional support of an expert/qualified/trained staff. (MW) | Being able to choose the best strategies to promote the well-being and conservation of individuals, groups, and species; being able to choose the best strategies to promote educational activities; being able to work independently (in terms of space, instruments, skills, and education) and respecting the law; to be always updated on the new research about animal welfare, conservation, education the current legislation. (MA) | Equal right to professional practice; equal access to funds to develop and grow; to be able to have a clear and adequate legislation that protects at the work-place; being recognized in their role in the fulfilling of the mission statement of the zoo, in terms of welfare, conservation and education. (MF) |
| VETERINARY STAFF | Having an economically rewarding and comfortable job; working in a safe and secure environment; having the opportunity to guarantee the physiological and psychological welfare of the animals housed in the zoo; having the opportunity to self-realization and personal fulfillment; (VSW) | Being able to work independently (spaces, instruments, skills, and education) and in total respect of the laws; being able to participate in scientific projects and for the protection of nature as a whole; to have the possibility of being able to fulfil the ethical code of the profession; being able to contribute to choose the best strategies to promote and maintain the well-being of individuals, groups, and species; to be able to make decisions about the health of the animals and feasibility of interactions when animals’ health and welfare issues are concerned; having the necessary resources to prevent and treat diseases, to eliminate or reduce pain, suffering, injury, and fear and to promote well-being of the animals involved. (VSA) | Respect for their job and professional skills; being recognized as an advocate for the well-being of the animals, especially the ones that are involved in the interactions. (VSF) |
| ZOO | Having the support and the approval of society and Institutions in order to carry out conservation projects; having access to funds and to a satisfactory income; to guarantee a safe environment for the visitors, the staff and the animals; to guarantee educational and enjoyable activities for the visitors. (ZW) | Being able to carry out their mission (education, conservation, research) and to maintain high standards of well-being of the hosted animals; being able to collaborate with all the stakeholders in order to be consistent and to adopt a transparent regulation; to be able to follow European guidelines that guarantee the homogeneity of actions and norms; to be able to be in contact with other institutions and facilities in order to be always updated. (ZA) | Having a mission statement and being able to fulfil it; to have the funds to develop and grow; being able to have a clear and adequate legislation and indications on the rules to be respected; having consistent, up-to-date and effective internal legislation and competent inspections by the authorities in order to guarantee the standards required; promoting equal educational and entertainment opportunities. (ZF) |
