# Supplementary material for: The Animal-Visitor Interaction Protocol (AVIP) for the assessment of Lemur catta walk-in enclosure in zoos
Source: PLoS One. 2022 Jul 28;17(7):e0271409. doi: 10.1371/journal.pone.0271409 (PMC9333233; doi:10.1371/journal.pone.0271409)
Supplement: S1 Appendix — Tables (Tables A–E) used to perform the animal welfare risk assessment. (DOCX) [file pone.0271409.s007.docx]

**S1 Appendix. Animal Welfare Risk Assessment.** Tables used to perform the Animal Welfare Risk Assessment

**Table A.** **Management Checklist: Staff Actions and Procedures.** Adapted from [42]

| **Management Checklist: Staff Actions and Procedures** | | | |
| --- | --- | --- | --- |
|  | **Keepers and Staff Supervising Animal-Visitors Interactions** | **YES** | **NO** |
|  | Did the staff inform visitors about the rules to be followed during the animal-visitors interaction? (e.g., how to touch the animals, not to smoke, drink and eat, act slowly, and do not yell, etc.) | ☐ | ☐ |
|  | Did the keeper monitor animal behaviour during the animal-visitors interaction? | ☐ | ☐ |
|  | Did the keeper monitor visitors’ behaviour during the animal-visitors interaction? | ☐ | ☐ |
|  | Did the keeper recommend the visitor to wash their hands before animal-visitors interaction? | ☐ | ☐ |
|  | Did the keeper cease any work activity in case a serious uncontrolled risk was identified? (Suspect of disease or signs of irritability or aggression). | ☐ | ☐ |
|  | Did the keeper have continuous training about biosecurity practices, zoonotic risk and appropriate practices to minimize these risks? | ☐ | ☐ |
|  | Was the keeper trained in how to report exposures, accidents, injuries, and illnesses? | ☐ | ☐ |
|  | Did the keeper have continuous training about the procedures to avoid animal escape? | ☐ | ☐ |
|  | Did the keeper have continuous training to recognize signs of health problems and stress in the animals held in the zoos? | ☐ | ☐ |
|  | Did the keeper maintain suitable standards of hygiene to minimize the risk of disease transmission? | ☐ | ☐ |
|  | Did the keeper assess and document the potential impact of each interactive experience on animal welfare and review it periodically? | ☐ | ☐ |
|  | Did the keeper check that the animals are free of lesions/illness and/or disease before and after each animal-visitors interaction? | ☐ | ☐ |
|  | **Veterinarians** | **YES** | **NO** |
|  | Are veterinarians involved in the management decisions about the species and the individuals that participate in the interactions? | ☐ | ☐ |
|  | Did veterinarians compile and follow a preventive, curative, and nutritional veterinary program? | ☐ | ☐ |
|  | Did the veterinarians perform zoonotic risk analyses? | ☐ | ☐ |
|  | Did the veterinarians make sure that the food administered during the interaction is part of a nutritional veterinary program? | ☐ | ☐ |
|  | Did the veterinarians update the clinical and pathological records? | ☐ | ☐ |
|  | **Enclosure Checklist: Design, Construction and Procedure** | **YES** | **NO** |
|  | Was an animal interaction area, such as an animal enclosure where visitors can touch the animals, clearly defined? | ☐ | ☐ |
|  | Were additional barriers present where the visitors pass to go into the enclosure (to avoid the escape of animals when the visitors enter)? | ☐ | ☐ |
|  | Was a protocol to avoid escape during animal-visitor interactions defined? | ☐ | ☐ |
|  | Were physical safety barriers present between the visitors and the animals during the interaction? | ☐ | ☐ |
|  | Were unauthorised access prevented? | ☐ | ☐ |
|  | Was the area of the enclosure where the interactions occurred well-ventilated? | ☐ | ☐ |
|  | Was the area of the enclosure where the interactions occurred at least partially shaded? | ☐ | ☐ |
|  | Did the area of the enclosure where the interactions occurred allow the animals to avoid the interaction, if they wished, without being followed by the public? | ☐ | ☐ |
|  | Did the housing of the animals during interactions allow all individuals to participate in the interaction if they wished? | ☐ | ☐ |
|  | Were signs present displaying visitors’ rules during interaction (not to smoke, eat, drink, proper hand washing, etc.)? | ☐ | ☐ |
|  | Was the enclosure designed to allow correct cleaning and disinfection? | ☐ | ☐ |
|  | Were hand-washing facilities available before accessing the interaction area? | ☐ | ☐ |
|  | Was antibacterial hand gel available before accessing the interaction area? | ☐ | ☐ |
|  | Were there limits to the number of participants per activity? | ☐ | ☐ |
|  | Has an appropriate keepers/visitors/animals ratio been defined? | ☐ | ☐ |
|  | Were there protocols for managing the biosecurity risks associated with visitor-animal interaction and emergency procedures? | ☐ | ☐ |
|  | Yes/No responses are provided according to the following criteria: “Yes” is assigned whenever the Step C working group observes the staff performing the action (e.g., informing the audience what not to do during the interaction) or if the results of the staff member's actions are evident (e.g., there were appropriate signs advising visitors not to smoke);”No” is assigned when the consequences of the not performance of the action are evident (e.g., antibacterial hand gels were not available). | | |

**Table B. Frequency of exposure (FE) categories.** Adapted from [42]

| **Categories** | **Description** | **Value** |
| --- | --- | --- |
| Negligible | Event very unlikely to happen, not expected to happen, and with <5% chance of happening | 1 |
| Low | Event not expected to happen, but it may occur with a probability ranging from 5% to 30% | 2 |
| Moderate | Event expected to happen with a probability ranging from 31% to 70% | 3 |
| High | Event that will occur with a probability >70% | 4 |

**Table C. Animal Welfare Indicators for scenario 1.** Adapted from [42]

| **Title** | | **Behavioural Observations** |
| --- | --- | --- |
| Scope | | Animal-based measure: *Lemur catta* |
| Sample size | | Five animals |
| Method description | | According to ethogram (other behaviours: escape attempts, avoidance behaviours) |
| **Classification (**Individual level) | | |
| 1 | No evidence of behaviours reflecting the worsening of subjective experiences; | |
| 2 | Evidence of behaviours indicating the worsening of subjective experiences. | |

**Table D. Animal Welfare Indicators for scenario 2.** Adapted from [42]

| **Title** | | **Skin Lesions** |
| --- | --- | --- |
| Scope | | Animal-based measure: *Lemur catta* |
| Sample size | | Five animals |
| Method description | | These injuries can be caused by an improper approach by the visitor.  Conduct an external visual examination before and after the interaction session.  The skin of the animals must not show injuries or abnormalities. |
| **Classification** (for each animal, the extent and severity of the lesion must be defined by assigning the following scores) | | |
| 1 | No obvious injuries; | |
| 2 | Injury involving a limited area of the skin, without compromising the deep layers; | |
| 3 | Large lesion, involving deeper layers and possibly aggravated by an inflammatory state, and other traumas. | |

**Table E. Animal Welfare Indicators for scenario 3.** Adapted from [42]

| **Title** | | **Zooanthroponosis** |
| --- | --- | --- |
| Scope | | Animal-based measure: *Lemur catta* |
| Sample size | | Five animals |
| Method description | | In-depth diagnostic tests following detection of symptoms attributable to infective or diffusive diseases to determine the causes and ascertain their anthropic origin. |
| **Classification** | | |
| 1 | No diagnosis of infectious disease; | |
| 2 | Diagnosis of an infectious disease presenting with impairment of general health, asthenia, anorexia, or involvement of a single organ, without severe complications; | |
| 3 | Situation of a serious infection that involved multiple organs or with severe complications. | |
