# Supplementary material for: The Animal-Visitor Interaction Protocol (AVIP) for the assessment of Lemur catta walk-in enclosure in zoos
Source: PLoS One. 2022 Jul 28;17(7):e0271409. doi: 10.1371/journal.pone.0271409 (PMC9333233; doi:10.1371/journal.pone.0271409)
Supplement: S2 Appendix — Tables (Tables A–F) used to perform the human risk assessment. (DOCX) [file pone.0271409.s008.docx]

**S2 Appendix. Human Risk Assessment.** Tables used to perform the Human Risk Assessment

**Table A. Management Checklist: Preventive and Protective Measures.** Adapted from [24]

| **Management Checklist: Preventive and protective measures** | |  |  |
| --- | --- | --- | --- |
|  | **General preventive security measures** | **YES** | **NO** |
|  | Did the keeper provide adequate information in the entrance transition zone focused on awareness about risks involved with animal contact and how to reduce risks? | ☐ | ☐ |
|  | Did the keeper inform visitors on the possibility to store their personal items in the safe area (where visitors can storage their personal items to prevent them from becoming contaminated)? | ☐ | ☐ |
|  | Did the keeper monitor animal behaviour during the animal-visitors interaction? | ☐ | ☐ |
|  | Did the keeper monitor visitors’ behaviour during the animal-visitors interaction? | ☐ | ☐ |
|  | Did the keeper stop any activity in case of a serious uncontrolled risk is identified? (Suspect of disease or signs of irritability or aggression). | ☐ | ☐ |
|  | Did the keeper have continuous training about biosecurity practices, zoonotic risk and appropriate practices to minimize these risks? | ☐ | ☐ |
|  | Did the keeper have the knowledge of how to report exposures, accidents or injuries? | ☐ | ☐ |
|  | Did the keeper have continuous training about the procedures to avoid animal escape? | ☐ | ☐ |
|  | Did the keeper have continuous training to recognize signs of health problems and stress in the animals held in the zoos? | ☐ | ☐ |
|  | Did the keeper check that the animals are free of lesions/illness and/or disease before and after each animal-visitors interaction? | ☐ | ☐ |
|  | Were unauthorised access prevented? | ☐ | ☐ |
|  | Were adequate signs present displaying visitors’ rules during interaction (not to smoke, eat, drink, proper hand washing, etc.)? | ☐ | ☐ |
|  | Were there limits to the number of participants per activity? | ☐ | ☐ |
|  | Had an appropriate keepers/visitors/animals ratio been defined? | ☐ | ☐ |
|  | Did it exist a restriction about the access of children under the age of 5 and mentally impaired visitors without a supervisor? | ☐ | ☐ |
|  | Was a protocol to avoid escape defined? | ☐ | ☐ |
|  | **General control measures of zoonotic risk: biosecurity** | **YES** | **NO** |
|  | Does a document describing all biosafety measures (specific for the exhibit considered) exist? | ☐ | ☐ |
|  | **General control measures of zoonotic risk: veterinary control** | **YES** | **NO** |
|  | Are veterinarians involved in management decisions about the species and individuals that participate in interactions? | ☐ | ☐ |
|  | Did veterinarians compile and follow a preventive, curative, and nutritional veterinary program? | ☐ | ☐ |
|  | Did the veterinarians perform zoonotic risk analyses? | ☐ | ☐ |
|  | Did the veterinarians update the clinical and pathological records? | ☐ | ☐ |
|  | **General control measures of zoonotic risk: environmental hygiene** | **YES** | **NO** |
|  | Did the keeper maintain suitable standards of hygiene to minimize the risk of disease transmission? | ☐ | ☐ |
|  | Were specific protocols or guidelines about the procedures of sanitation? | ☐ | ☐ |
|  | Were the visitor walk-ways cleaned daily? | ☐ | ☐ |
|  | Were the visitor walk-ways cleaned whenever visibly contaminated? | ☐ | ☐ |
|  | **General control measures of zoonotic risk: design of the exhibition areas** | **YES** | **NO** |
|  | Was a dedicated visitor entrance and exit with transition zones and a separate service access point and was isolated from the rest of the zoo? | ☐ | ☐ |
|  | Did the visitors flow on one way? | ☐ | ☐ |
|  | Was an animal interaction area, such as an animal enclosure where visitors can touch the animals, clearly defined? | ☐ | ☐ |
|  | Did it exist a service access point differentiate from visitors’ entrance or exit? | ☐ | ☐ |
|  | Does it exist a safe area (an area where visitors can store their personal items to prevent them from becoming contaminated)? | ☐ | ☐ |
|  | Were additional barriers present where the visitors pass to go into the enclosure (to avoid the escape of animals when the visitors enter)? | ☐ | ☐ |
|  | Were physical safety barriers present between the visitors and the animals during the interaction? | ☐ | ☐ |
|  | Was the area of the enclosure where the interactions occurred well-ventilated? | ☐ | ☐ |
|  | Was the enclosure designed to allow correct cleaning and disinfection? | ☐ | ☐ |
|  | Were visitor walk-ways through the interaction designed to allow for effective cleaning and disinfection? | ☐ | ☐ |
|  | Does it exist an appropriate number of hand-washing stations accessible to all visitors regardless of age or height? | ☐ | ☐ |
|  | **General control measures of zoonotic risk: control measures for the risks of infection** | **YES** | **NO** |
|  | Did the keeper inform visitors about the rules to be followed during the animal-visitors interaction? (e.g., how to touch the animals, not to smoke, drink and eat, act slowly, and do not yell, etc.) | ☐ | ☐ |
|  | Did the keeper recommend the visitor to wash their hands before animal-visitors interaction? | ☐ | ☐ |
|  | Did the keeper recommend the visitor to wash their hands after animal-visitors interaction? | ☐ | ☐ |
|  | Were hand-washing stations, wipes or antimicrobial gels available before accessing the interaction area? | ☐ | ☐ |
|  | Were hand-washing stations, wipes or antimicrobial gels available after accessing the interaction area? | ☐ | ☐ |
|  | Did the automatic (or foot-operated) washing stations have a sufficient volume of water flow, soap dispenser, paper towel dispensers and trash bins? | ☐ | ☐ |
|  | Did the keeper inform visitors about the non-edible nature of the food (if the products are provided to visitors to feed the animals)? | ☐ | ☐ |
|  | **Protective measures: *Personal Protective Equipment* (*PPE)*** | **YES** | **NO** |
|  | Are visitors provided with PPE to reduce any risk of contamination? | ☐ | ☐ |
|  | Are visitors provided with PPE to reduce any risk of injuries? | ☐ | ☐ |

**Table B. List of relevant zoonosis in Lemurs and in other animals reported in zoo human–animal interactions included in Step C - Phase 1 and relative bibliography.**

| **Disease** | **Species** | **Reference** |
| --- | --- | --- |
| Tuberculosis | *Mycobacterium tubercolosis*e; *M. bovis* | [1-4] |
|  | *Klebsiella pneumoniae* |  |
| *E.coli* infections | *Escherichia coli* | [5-10] |
| Salmonellosis | *Salmonella enterica* | [5-7, 10] |
|  | *Shigella* spp | [5] |
|  | *Vibrio* cholerae | [5] |
| Yersiniosis | *Yersinia pseudotuberculosis, Y. enterocolitica* | [5-11] |
| Campylobacteriosis | *Campylobacter fetus* subsp *jejuni* | [12, 13] |
|  | *Leptospira* spp. | [14-16] |
|  | *Francisella tularensis* | [17] |
| Antimicrobial-Resistant bacteria | Methicillin resistant S. aureus (MRSA),  Extended spectrum beta-lactamase (ESBL) | [18-21] |
| Rabies | Rabies lyssavirus | [22] |
|  | *Lymphocryptovirus* | [6, 7] |
| Cryptosporidiosis | *Cryptosporidium* spp. | [23] |
|  | *Giardia duodenalis* | [23, 24] |
|  | *Entamoeba* sp. | [24] |
|  | *Encephalitozoon cunicoli, E.intestinalis; E. bieneusi* | [25-28] |
| Dermatophytosis | *Trichophyton mentagrophytes* | [28] |

**Table C. Probability of people being exposed to a specific hazard – categories established considering the existing preventive measures.** Adapted from [24]

| **Categories** | **Description** | **Value** |
| --- | --- | --- |
| Rare | Event very unlikely to happen with <5% chance of it happening | 1 |
| Unlikely | Event not expected to happen, but it may present with a probability ranging from 10% to 30% | 2 |
| Probable | Expected to happen with a probability ranging from 30% to 95% | 3 |
| Very likely | Event that will occur with a probability >95% | 4 |

**Table D. Extent of the damage – categories established to represent the extent of the damage, considering the existing protective measures.** Adapted from [24]

| **Categories** | **Description** | **Value** |
| --- | --- | --- |
| Minor | Minor damage that can be solved with medical treatment on-site (first aid kit) | 1 |
| Moderate | Moderate damage requiring at least a few days prognosis | 2 |
| Serious injuries | Damage requiring more than 30 days prognosis or that could be permanent | 3 |
| Very likely | Unfavorable prognosis | 4 |

**Table E. Risk calculation.** Adapted from [24]

| **Risk = P x D** | | | **Hazard Characterization (P)** | | | |
| --- | --- | --- | --- | --- | --- | --- |
|  |  |  | **Rare** | **Unlikely** | **Probable** | **Very Likely** |
|  |  |  | 1 | 2 | 3 | 4 |
| **Exposure Assessment (D)** | **Death** | 4 | 4 | 8 | 12 | 16 |
|  | **Serious** | 3 | 3 | 6 | 9 | 12 |
|  | **Mild** | 2 | 2 | 4 | 6 | 8 |
|  | **Lower** | 1 | 1 | 2 | 3 | 4 |

**Table F. Risk categories.** Adapted from [24]

| **Type of Risk** | **R value** | **Action Required** |
| --- | --- | --- |
| Low Risk (L) | R < 4 | No action required |
| Moderate risk (M) | 4 ≤ R < 9 | Medium-term action (within 1 year) |
| High risk (H) | 9 ≤ R ≤ 12 | Urgent action (within 3 months) |
| Very high risk (V) | R > 12 | Immediate action |

**References**

1. Knezevic AL, McNulty WP. Tubercolosis in Lemur Mongoz. Folia Primatol. 1967;6:153–159.
2. Wilson P, Weavers E, West B, Taylor M, Kavanagh J, Jones P. *Mycobacterium bovis* infection in primates in Dublin Zoo: epidemiological aspects and implications for management. Lab Anim. 1984;18:383–7.
3. Richard C. Epidemiology of Klebsiella pneumoniae infections in 2 colonies of squirrel monkeys and lemurs. Bull Soc Pathol Exot Filiales. 1989;82:458–464.
4. Sasaki E, Tokiwa T, Tsugo K, Higashi Y, Hori H, Une Y. Peracute Bacterial Meningitis due to Infection with Klebsiella pneumoniae in Captive-bred Ruffed Lemurs (*Varecia variegate*). J Comp Pathol. 2017;156:281–285.
5. Bublitz DC, Wright PC, Rasambainarivo FT, Arrigo-Nelson SJ, Bodager JR, Gillespie TR. Pathogenic enterobacteria in lemurs associated with anthropogenic disturbance. Am J Primatol. 2015;77:330–337.
6. Poli G, Cocilovo A, Dell’Ara PE, Martino PA, Ponti W. Microbiologia e immunologia veterinaria. Microbiologia e immunologia veterinaria. UTET Scienze mediche; 2005.
7. Bauerfeind R, von Graevenitz A, Kimmig P, Schiefer HG, Schwarz T, Slenczka W, Zahner H. Zoonoses: Infectious Diseases Transmissible Between Animals and Humans; Washington (USA): ASM Press; 2015.
8. Miller RE, Fowler ME. Fowler's Zoo and Wild Animal Medicine, Volume 8. St Louis, MO: Saunders Elsevier; 2014
9. Hugh-Jones ME, Hubbert WT, Hagstad HV. Zoonoses: recognition, control, and prevention. Iowa State University Press, Ames, Iowa; 1995
10. Calle PP, Ott Joslin J. New world and old world monkeys. In Fowler’s Zoo and Wild Animal Medicine; Miller E, Fowler M editors; Amsterdam: Elsevier Inc; 2015;
11. Bresnahan JF, Whitworth UG, Hayes Y, Summers E, Pollock J. Yersinia enterocolitica infection in breeding colonies of ruffed lemurs. J Am Vet Med Assoc. 1984;185:1354–6.
12. Villers LM, Jang SS, Lent CL, Lewin-Koh SC, Norosoarinaivo JA. Survey and comparison of major intestinal flora in captive and wild ring-tailed lemur (*Lemur catta*) populations. Am J Primatol. 2008;70:175–184.
13. Luechtefeld NW, Wang WL. Campylobacter fetus subsp. jejuni in a turkey processing plant. J Clin Microbiol. 1981;13:266–8.
14. Desvars A, Naze F, Vourc’h G, Cardinale E, Picardeau M, Michault A, et al. Similarities in Leptospira serogroup and species distribution in animals and humans in the Indian ocean island of Mayotte. Am J Trop Med Hyg. 2012;87:134–140.
15. Ferreira AS, Ahmed A, Rocha T, Vieira ML, Paiva-Cardoso M, das N, et al. Genetic diversity of pathogenic leptospires from wild, domestic and captive host species in Portugal. Transbound Emerg Dis. 2019;67:852–864.
16. Thanaraj V, Topping K, Cheesbrough J, Woywodt A. A day in the zoo. Clin Kidney J. 2014;7(3):318-319.
17. Calle PP, Bowerman DL, Pape WJ. Nonhuman Primate Tularemia (*Francisella tularensis*) Epizootic in a Zoological Park. J Zoo Wildl Med. 1993:459–468.
18. Heaton CJ, Gerbig GR, Sensius LD, Patel V, Smith TC. *Staphylococcus aureus* Epidemiology in Wildlife: A Systematic Review. Antibiotics. 2020;9:89
19. Schaumburg F, Mugisha L, Kappeller P, Fichtel C, Köck R, Köndgen S, et al. Evaluation of non-invasive biological samples to monitor *Staphylococcus aureus* colonization in great apes and lemurs. PLoS ONE. 2013;8:e78046
20. Springer A, Mellmann A, Fichtel C, Kappeler PM. Social structure and *Escherichia coli* sharing in a group-living wild primate, Verreaux’s sifaka. BMC Ecol. 2016;16:1–12.
21. Soge OO, No D, Michael KE, Dankoff J, Lane J, Vogel K, et al. Transmission of MDR MRSA between primates, their environment and personnel at a United States primate centre. J. Antimicrob. Chemother. 2016;71:2798–2803.
22. Reuter KE, Clarke TA, LaFleur M, Schaefer MS. Rabies in primates: are aggressive pet lemurs a risk to humans? Madagascar Conserv Dev. 2018;13:53.
23. Rasambainarivo FT, Gillespie TR, Wright PC, Arsenault J, Villeneuve A, Phane Lair S. Survey of Giardia and Cryptosporidium in lemurs from the Ranomafana National Park, Madagascar. J Wildl Dis. 2013;49:741–743.
24. Berrilli F, Prisco C, Friedrich K, Di Cerbo P, Di Cave D, De Liberato D. *Giardia duodenalis* assemblages and Entamoeba species infecting non-human primates in an Italian zoological garden: Zoonotic potential and management traits. Parasites Vectors. 2011;4:1–8
25. Yabsley MJ, Jordan CN, Mitchell SM, Norton TM, Lindsay DS. Seroprevalence of *Toxoplasma gondii*, S*arcocystis neurona*, and *Encephalitozoon cuniculi* in three species of lemurs from St. Catherines Island, GA, USA. Vet Parasitol. 2007;144:28–32.
26. Li W, Deng L, Yu X, Zhong Z, Wang Q, Liu X, et al. Multilocus genotypes and broad host-range of *Enterocytozoon bieneusi* in captive wildlife at zoological gardens in China. Parasit. Vectors. 2016;9:1–9.
27. Słodkowicz-Kowalska A, Majewska AC, Trzesowska E, Skrzypczak L. Occurrence of Encephalitozoon intestinalis in the Red ruffed lemur (*Varecia rubra*) and the Ring-tailed lemur (*Lemur catta*) housed in the Poznan Zoological Garden, Poland. Ann Parasitol. 2012;58:49–52.
28. Phair K, Larsen RS, Wack R. Dermatophytosis (*Trichophyton mentagrophytes*) in a Coquerel’s Sifaka (*Propithecus coquereli*). J Zoo Wildl Med. 2011;42:759–762.
